# Sex- and region-specific differences in microstructural remodeling and passive biomechanics of the aorta correlate with aneurysm propensity in a mouse model of severe Marfan syndrome

**DOI:** 10.1016/j.actbio.2025.05.056

**Published:** 2025-05-23

**Authors:** Krashn Kumar Dwivedi, Yufan Wu, Jacob Rother, Jessica E. Wagenseil

**Affiliations:** Department of Mechanical Engineering and Materials Science, Washington University in St. Louis, St. Louis, MO, United States

**Keywords:** Marfan syndrome, *Fbn1* ^mgR/mgR^, Sex, Aortic aneurysm, ECM remodeling, Passive biomechanics

## Abstract

Marfan syndrome (MFS) is a connective tissue disorder caused by mutations in the gene that encodes fibrillin-1, a glycoprotein necessary for elastic fiber assembly and stability in the large elastic arteries. MFS is associated with aortic aneurysms that typically occur in the proximal ascending aorta and have worse outcomes in males. Mechanisms for the sex- and region-specific differences in aneurysm development and outcomes are unknown. We quantified aortic geometry, microstructural remodeling, and passive biomechanics of the thoracic ascending, thoracic descending, abdominal suprarenal, and abdominal infrarenal aorta in 4 months old male and female *Fbn1*^*mgR/mgR*^ (a model of severe MFS) and littermate wild-type mice to determine correlations between aortic geometry, microstructural remodeling, biomechanics, and aneurysmal dilation. We showed that aneurysmal dilation was strongly correlated with unloaded thickness, microstructural remodeling including loss of elastic fibers, deposition of collagen fibers, and decrease in cell nuclei number, and mechanical metrics including physiologic and ex vivo circumferential material stiffness. A multivariable mixed model showed that unloaded thickness, elastic fiber degradation, and ex vivo material stiffness predicted aneurysmal dilation with an adjusted R^2^ = 0.8818. Our results highlight the potential of geometric, microstructural remodeling, and biomechanical metrics to serve as physical biomarkers for personalized aortic aneurysm diagnosis and management in MFS. *Statement of significance:* Marfan syndrome (MFS) is a genetic disease associated with aortic aneurysms that have distinct sex- and region-specific outcomes. The mechanisms driving these variations are unclear. We used a severe MFS mouse model (*Fbn1*^*mgR/mgR*^) to explore differences in microstructural remodeling and passive wall mechanics along the aortic length in males and females. We correlated these changes with aneurysm severity, as quantified by aortic dilation. We found that sex- and region-specific alterations in unloaded thickness, microstructural remodeling, and passive mechanical properties of the aortic wall play a critical role in aortic dilation. Our findings showed that mechanical metrics, particularly ex vivo material stiffness, may serve as biomarkers for the diagnosis and management of aortic aneurysms.

## 1. Introduction

Marfan syndrome (MFS) is a congenital connective tissue disorder associated with a range of systemic complications affecting the cardiovascular system, among other organs. It is inherited as an autosomal dominant condition with mutations in the fibrillin-1 (*FBN1*) gene [1]. The *FBN1* gene encodes a glycoprotein essential for maintaining the long-term structural integrity and stability of elastic fibers within the large elastic arteries [2]. Mutations in *FBN1* trigger changes in the aortic wall that include progressive extracellular matrix (ECM) remodeling, smooth muscle cell (SMC) phenotype modulation and/or apoptosis, and activation of transforming growth factor-beta (TGF-β) signaling [3–6]. This disease process in MFS leads to alterations in the mechanical properties of the aortic wall, collectively weakening the wall and contributing to the development, progression, and eventual dissection and/or rupture of aortic aneurysms [2,7–10]. Clinical management of aneurysms in MFS involves regular imaging to monitor aortic size and growth rate to assess the risk of aortic dissection and/or rupture [11,12]. However, these criteria fail to provide precise guidance for sex-specific aneurysm management, particularly in the context of genetic disorders such as MFS [13–15], therefore other physical biomarkers are recommended [13,16,17].

Although the prevalence of MFS is independent of sex, epidemiological evidence suggests that males with MFS experience more severe aortic complications, including incidence of aneurysm development, aortic surgery, and aortic dissection, compared to females [18,19]. The mechanisms underlying this observed sexual dimorphism are unclear. Hormonal differences, particularly involving sex hormones, are believed to play a crucial role in the differing outcomes of MFS between males and females [20,21]. Many previously reported studies on mouse models of MFS have largely been confined to a single sex (male). Studies that have included males and females demonstrate sex dependent ECM remodeling [22], SMC phenotype modulation and contractility [7,22], and androgen stimulated TGF-β activity [20] in a mouse model of mild MFS (*Fbn1*^*C1041G/*+^). Our previous study [8] using a severe mouse model of MFS (*Fbn1*^*mgR/mgR*^) demonstrated that differences in ECM remodeling and passive wall biomechanics between males and females played a critical role in sex-specific ascending aortic aneurysm development and progression, but we did not look at multiple aortic regions.

While MFS-associated aortic aneurysms can theoretically affect the entire aorta, clinical and experimental evidence indicates that aneurysmal expansion predominantly originates at the aortic root and remains localized to the ascending aorta, with limited progression in the descending or abdominal aorta [7,23–26]. The underlying mechanisms behind this region-specific aneurysm development remain poorly understood but are thought to involve differences in embryonic lineage of cells [27,28], the extent of ECM remodeling [29], SMC dropout [27,30], and physiologic loading conditions [31,32] along the length of the aorta. Recent studies in mouse models of MFS reveal widespread mechanical abnormalities throughout the aorta [7,9,23]. However, the degree of ECM remodeling and mechanical dysfunction is markedly more severe in the ascending compared to the descending aorta, correlating with its heightened susceptibility to aneurysmal degeneration [9,23]. The effect of MFS pathology on aortic wall biomechanics is region-specific in humans, as well, but can change with disease progression [33,34]. Changes in the geometry and mechanical behavior along the aortic length can affect the timing and location of reflected pressure pulse waves altering local mechanical stresses on the aortic wall [35], hence it is important to understand MFS-associated remodeling along the entire aortic length.

The aim of the current study was to investigate sex-specific effects of MFS on microstructural remodeling and passive biomechanics along the length of the mouse aorta to identify physical biomarkers for aneurysmal dilation that may be related to the incidence of aortic events. Geometric and biomechanical data under loaded conditions, along with microstructural remodeling under unloaded conditions, were collected for the thoracic ascending aorta, thoracic descending aorta, abdominal suprarenal aorta, and abdominal infrarenal aorta from *Fbn1*^*mgR/mgR*^ and wild-type, male and female mice at 4 months of age. The contributions of unloaded geometry, microstructural remodeling, and passive mechanical properties to aortic dilation were analyzed using a multivariable mixed model to identify physical biomarkers. Our results highlight the potential of geometric, microstructural remodeling, and biomechanical metrics to serve as physical biomarkers for personalized aortic aneurysm diagnosis and management in MFS.

## 2. Materials and methods

### 2.1. Mice

All protocols were approved by Washington University Institutional Animal Care and Use Committee and conform to the current NIH guidelines. A severe mouse model of MFS (*Fbn1*^*mgR/mgR*^ = MU) [36]) and wild-type (WT) littermates were used to investigate the sex- and region-specific outcomes of reduced fibrillin-1 amounts on aortic dilation, microstructural remodeling, and passive wall mechanics. MU and WT mice were obtained from breeding pairs of *Fbn1*^*mgR/*+^male and female mice. Mice were backcrossed > 10 generations onto a C57BL6J background and refreshed by backcrossing every three generations. Data were collected from thoracic (ascending (ASC) and descending (DSC)) and abdominal (suprarenal (SAB) and infrarenal (IAB)) regions of the aorta from 4 months old WT and MU, male and female mice with 5–12 mice/group for a total of 37 mice. Supp. Table S1 presents the numbers of samples per group used for each measurement. Data from the ASC has been previously published [8].

### 2.2. Blood pressure and tissue collection

After blood pressure measurement [8,37] (Table 1), the mouse was euthanized by CO_2_ inhalation in a Smart Box. The whole aorta was isolated and segmented into four regions: ASC (between aortic root and right coronary artery), DSC (between the first intercostal artery and diaphragm), SAB (between the left gastric artery and left renal artery) and IAB (between the right renal artery and bifurcation of iliac artery). All aortic segments were carefully washed in phosphate buffer saline (PBS), stored in PBS at 4 °C, and mechanically tested within 3 days [38]. Unloaded dimensions and residual stretch were measured after mechanical testing and then a subset of the segments was processed for imaging.

### 2.3. Microstructural imaging and quantification

#### 2.3.1. Aortic wall imaging

Multiphoton microscopy was used for imaging and quantification of microstructure of aortic wall as described in our previous study [8]. Briefly, aortic segments were fixed in 4 % paraformaldehyde overnight, washed in PBS, and stored in 70 % ethanol at 4 °C. For en face imaging, the aortic segments were cut open and mounted on glass slides with DAPI gold mounting media with the circumferential and axial directions aligned with the long and short edges of the glass slide, respectively. Z-stacks of ASC, DSC, SAB and IAB were collected using a sp-8 DIVE multiphoton microscope (Leica) with a 60× objective and 2 μm step sizes. Collagen fibers, elastic fibers, and cell nuclei were excited at 880 nm and imaged using second harmonic generation (SHG) (emission: 420–460 nm), autofluorescence (emission: 495–540 nm), and DAPI (emission: 430–450 nm) [8,39,40], respectively. Images were obtained for 5–6 aortic segments/group (Supp. Table 1).

#### 2.3.2. Quantification of wall microstructure

Elastic fiber porosity, collagen fiber amounts, tortuosity, and thickness, and cell nuclei number and aspect ratio, were determined as previously described [8,41] and summarized in Supp. Fig. S1. The medial and adventitial layers were defined by the relative intensity of the collagen fiber images throughout the image stack (Supp. Fig. S1A). Elastic fiber porosity was defined as the ratio of the area of holes to the total area covered by elastic fibers within the medial (middle) layer (Supp. Fig. S1B). The volumetric content, tortuosity (total fiber length/end to end length), and thickness of collagen fibers (Supp. Fig. S1C) were quantified within the medial and adventitial layers. The number of cell nuclei and the nuclei aspect ratio (major/minor axes of fitted ellipse) (Supp. Fig. S1D) were quantified for the medial layer.

### 2.4. Passive mechanical testing

Ex vivo, passive mechanical testing on each aortic segment was performed as previously reported [8,42]. Briefly, ASC, DSC, SAB and IAB were submerged in PBS at 37 °C, mounted on custom stainless steel cannulae in a pressure myograph (110P, Danish Myotechnology), and secured using 7.0 surgical sutures. The unloaded length (*L*) of the aortic segment was determined as the length at which there was no change in force when displacing the micrometer by ±10 μm. The relative physiologic length was defined by the loaded length (*l*) at which there was minimal force change (±3 mN) when the aortic segment was inflated from 0 to 150 mmHg [43]. The physiologic axial stretch ratio was defined as *l/L*. For preconditioning, the aortic segment was held at the physiologic axial stretch ratio and cyclically inflated three times from 0 to 150 mmHg at a constant rate of 3 mmHg/sec. The aortic segment was then subjected to three inflation cycles between 0 and 150 mmHg in increments of 15 mmHg at a rate of 3 mmHg/sec between steps and a hold time of 5 s/step. Pressure, outer diameter and axial force were recorded at 1 Hz. The aortic segment was removed from the cannulae and three rings were cut and imaged to obtain the unloaded dimensions (inner diameter and thickness) (Supp. Fig. 2A). The average unloaded dimensions from three aortic rings were calculated and used for stress and stretch calculations for each sample. The rings were then radially cut and imaged to measure the circumferential residual stretch (Supp. Fig. S2B) [44]. Unloaded geometry and mechanical testing data were obtained for 5–12 aortic segments/group (Supp. Table 1).

#### 2.4.1. Physiologic circumferential stiffnesses

The deformed outer diameters (*d*_*out*_) corresponding to each applied pressure step were determined from the recorded data. Outer diameters corresponding to systolic, diastolic, and mean pressures for each animal (averages for each group provided in Table 1) were calculated by interpolation of the pressure-outer diameter data [8,42]. The corresponding deformed inner diameters (*d*_*in*_) were determined assuming incompressibility of the aortic wall. We calculated two values of circumferential stiffness: Peterson’s modulus (*E*_*p*_) (a measure of structural stiffness) and incremental Young’s modulus (*E*_*i*nc_) (a measure of material stiffness) [45],

(1a.b) $$E_{p}=\frac{d_{dia,in}\times pp}{d_{sys,\;in}-d_{dia,in}},\;E_{inc}=\frac{d_{dia,in}^{2}\times pp}{2\;t_{dia}\;\left(d_{sys,in}-d_{dia,in}\right)},$$

where *d*_dia,in_ and *d*_sys,in_ are the inner diameters at diastolic (dia) and systolic (sys) pressures, *t*_dia_ is the thickness at diastolic pressure, and *pp* is the pulse pressure (averages for each group provided in Table 1).

#### 2.4.2. Physiologic stretch ratios and stresses

Physiologic stretch ratios and stresses were calculated at animal specific mean blood pressures (averages for each group provided in Table 1). The physiologic circumferential stretch ratio (*λ*_θ_) was calculated as the ratio of deformed diameters (*d*_*out*_ and *d*_*in*_) at the mean blood pressure to undeformed diameters (*D*_*out*_ and *D*_*in*_). The physiologic axial stretch ratio (*λ*_z_) was defined as the ratio of physiologic deformed length (*l*) to unloaded length (*L*) of each aortic segment.

(2a.b) $$\lambda_{\theta }=\frac{1}{2}\left(\frac{d_{in}}{D_{in}}+\frac{d_{out}}{D_{out}}\right),\;\lambda_{z}=\frac{l}{L}.$$

Assuming no shear, the average physiologic axial (*σ*_*z*_) and circumferential (*σ*_*θ*_) wall stresses were calculated by,

(3a.b) $$\sigma_{\theta }=\frac{p\;d_{in}}{d_{out}-d_{in}},\;\sigma_{z}=\frac{4f+p\;d_{in}^{2}}{\pi \left(d_{out}^{2}-d_{in}^{2}\right)},$$

where *f* is the measured axial force and all quantities were calculated at the animal specific mean blood pressure.

#### 2.4.3. Ex vivo circumferential material stiffnesses

The circumferential stretch-stress curve for the aorta shows a nonlinear response that can be described by two linear functions. The slopes of the linear fits in the lower and higher stretch regions are commonly attributed to the ex vivo circumferential material stiffness of the aortic wall provided by elastic and collagen fibers, respectively [46, 47]. These “low” and “high” moduli were obtained by linear fits of the experimental stretch-stress curves in the lower (up to 60 mmHg) and the higher (120–150 mmHg) stretch regions using custom Matlab (Mathworks) scripts [8] (Supp. Fig S3).

### 2.5. Statistical analyses

Statistical analyses were either conducted using GraphPad (Prism) or R-studio (Posit, PBC). Outliers were excluded using the Rout test. The numbers of samples included for each measurement for each group are shown in Supp. Table S1. Statistical significance was set with 95 % confidence (*p* < 0.05). Two-way ANOVA was used to determine the effects of sex (S) and genotype (G) and their interaction for each aortic segment (ASC, DSC, SAB and IAB) on microstructural remodeling, unloaded geometry, and passive biomechanics. Tukey–Kramer posthoc test identified differences between specific pairs, focusing on differences between genotypes for the same sex for each aortic region. Percent variation attributed to sex, genotype, and sex/genotype interaction and significant p values are reported in the text or figures.

Pearson correlation with linear regression analyses were performed between metrics for each aortic segment and for all aortic segments combined to identify significant correlations between metrics. Pearson correlation with linear regression analyses were also performed between metrics and the loaded inner diameter at average mean blood pressure for all aortic segments combined. These analyses identified individual metrics that had strong correlations with the clinical metric (aortic diameter) used for managing aneurysms associated with MFS. Mixed multivariable regression models were then constructed using forward stepwise procedures to examine the ability of multiple metrics to predict the aortic dilation (aortic inner diameter at mean pressure) for all aortic segments combined. We examined three scenarios based on the type of metric: in case 1, the possible predictors were microstructural remodeling metrics; in case 2, the possible predictors were mechanical metrics; and in case 3, microstructural remodeling metrics, mechanical metrics, and wall unloaded dimensions were combined as possible predictors. All possible combinations of predictors were tested in each case. Multivariable models with high multicolinearity (Variance Inflation Factor ≥ 5) and low significance (*p* ≥ 0.05) were rejected [48]. The “best” model in each case was chosen by the highest adjusted R^2^. For the correlation analyses and multivariable regression models, samples were only included if they had appropriately matched data (i.e. all microstructural and mechanical metrics for the same sample). A total of 78 samples for all aortic segments had appropriately matched data.

## 3. Results

### 3.1. Blood pressures

The blood pressures for WT and MU, male and female mice were previously published [8]. Animal-specific blood pressures were used to calculate the mechanical metrics. The averages for each group are presented in Table 1.

### 3.2. Unloaded dimensions and circumferential residual stretches

**Fig. 1.**
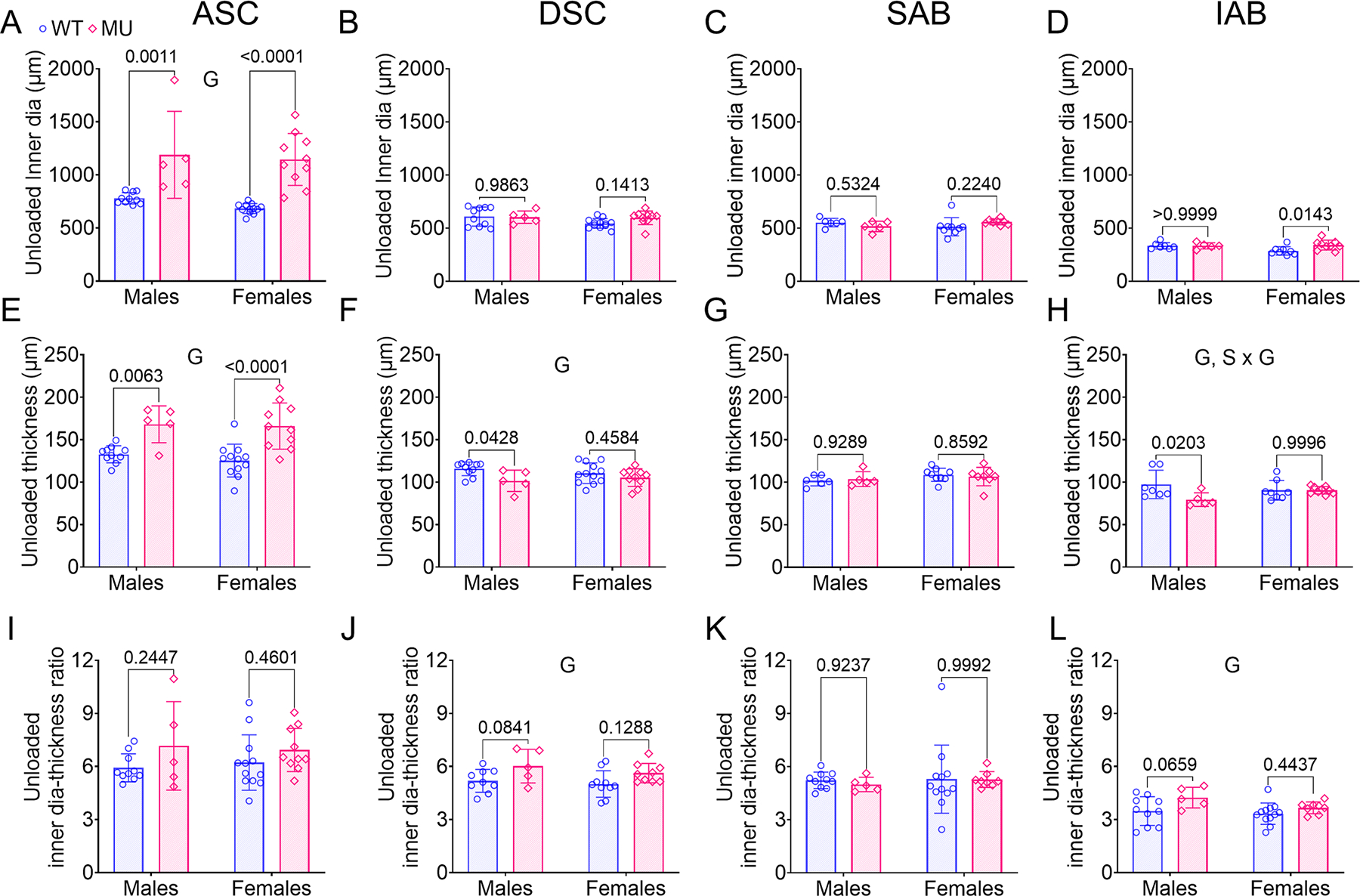
Unloaded inner diameter (A–D), thickness (E-H), and diameter-thickness ratio (I-L) of ASC, DSC, SAB and IAB for each group. The unloaded dimensions were measured from aortic rings (Supp. Fig. S2A). Letters indicate significant effects by two-way ANOVA for the independent variables (sex (S) and genotype (G)). *P* values indicate significant difference between genotypes for each sex by Tukey’s post hoc test. Individual data points and mean ± SD are shown. *N* = 5 – 12/group. WT = blue circles, MU = pink diamonds.

The unloaded inner diameter and thickness were measured from image analyses of cut rings (Supp. Fig. S2A). Unloaded inner diameter, thickness, and inner diameter to thickness ratio for ASC, DSC, SAB and IAB are shown in Fig. 1. The unloaded inner diameter and thickness of ASC depended on genotype (53 % variation, *p* < 0.0001 for inner diameter and 45 % variation, *p* < 0.0001 for thickness), with both dimensions being significantly larger in MU male and female ASC compared to WT (Fig. 1A, E). The unloaded thickness of DSC depended on genotype (17 % variation, *p* = 0.0139) (Fig. 1F). The unloaded thickness of IAB depended on genotype (14 % variation, *p* = 0.0439) and the interaction between sex and genotype (14 % variation, *p* = 0.0409) (Fig. 1H). For the DSC and IAB, the MU unloaded thickness was smaller than WT, which was opposite to the unloaded thickness changes in MU ASC compared to WT ASC. The unloaded inner diameter of DSC (Fig. 1B), SAB (Fig. 1C), and IAB (Fig. 1D) and the unloaded thickness of SAB (Fig. 1G) did not significantly depend on genotype, sex, or their interaction. The inner diameter to unloaded thickness ratios for DSC and IAB depended on genotype (Fig. 1J, L) (22 % variation, *p* = 0.0081 for DSC and 15 % variation, *p* = 0.0200 for IAB), with no significant differences for the ASC (Fig. 1I) or SAB (Fig. 1K). These findings demonstrate ASC-specific increases in unloaded diameter and thickness in MU mice compared to WT.

The inner and outer residual stretches were quantified from images of radially opened aortic rings (Supp. Fig. S2B) and compared between groups (Supp. Fig. S4). Our findings showed that the intimal layer was under compressive stretch (inner residual stretch < 1), while the adventitial layer was under tensile stretch (outer residual stretch > 1), and the residual stretch ratios were relatively consistent for all regions of the aorta. The inner residual stretch ratios of the ASC and DSC were greater in MU mice compared to WT (41 % variation, *p* = 0.0005 for the ASC and 15 % variation, *p* = 0.0297 for the DSC) (Supp. Figs. S4A, B). The inner residual stretch ratios of the SAB and IAB were similar between groups (Supp. Figs. S4C, D). The outer residual stretch of the ASC was greater in MU mice compared to WT (18 % variation, *p* = 0.0269) (Supp. Fig. S4E) and there was an interaction between sex and genotype in the IAB (26 % variation, *p* = 0.0233) (Supp. Fig. S4H). There were no significant differences between groups for the outer residual stretch ratios of the DSC and SAB (Supp. Figs. S4F, G). These findings demonstrate a decrease in compressive stretch in the intimal layer and an increase in tensile stretch in the adventitial layer that is specific to the MU ASC.

### 3.3. Microstructural remodeling

**Fig. 2.**
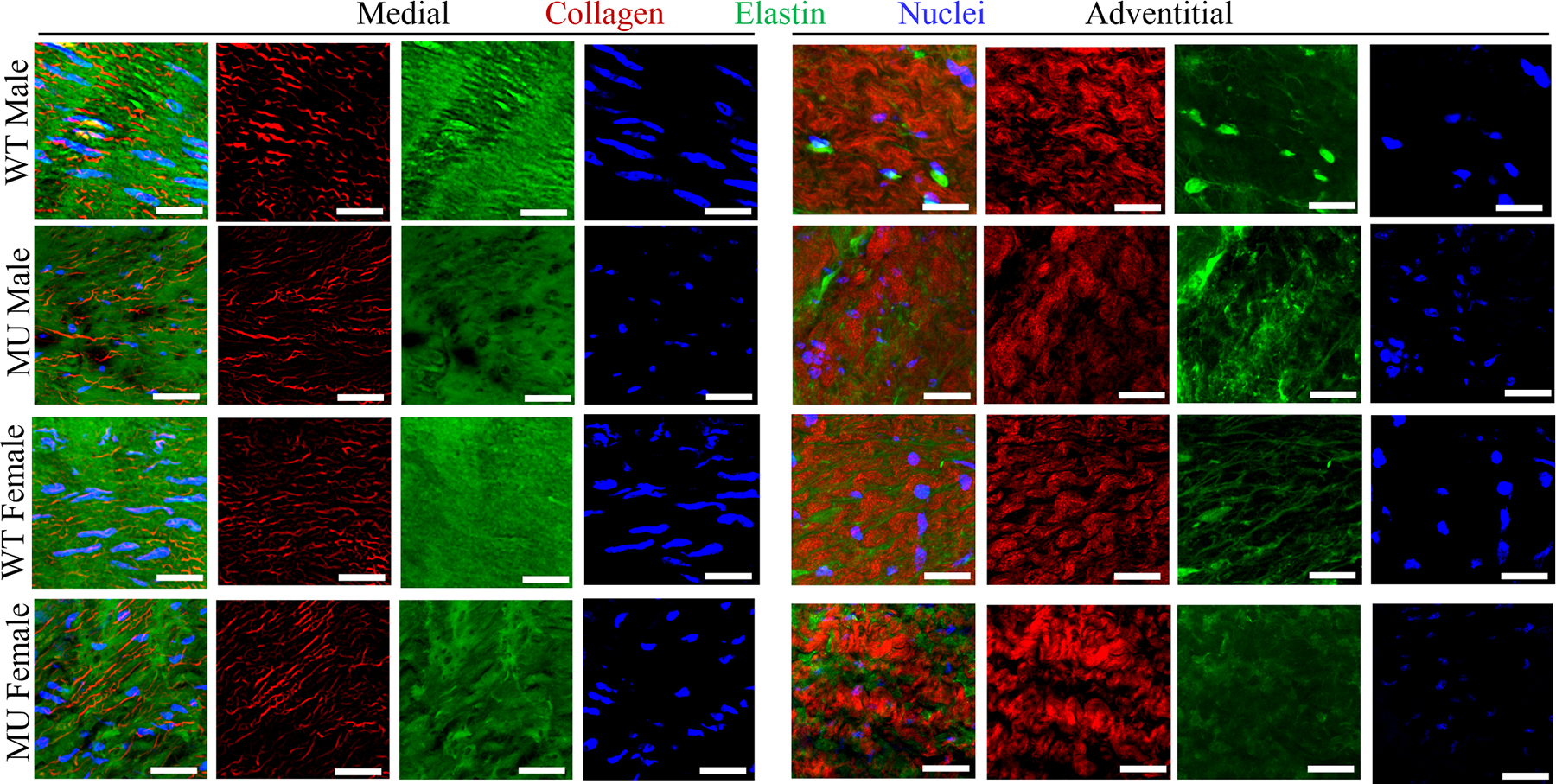
Representative en face multiphoton images of ASC for WT and MU, male and female mice. These images illustrate the qualitative changes in collagen fiber (red), elastic fiber (green), and cell nuclei (blue) organization in the medial and adventitial layers for each group. The multiphoton images for DSC, SAB and IAB are presented in Supp. Figs. S5–S7. Scale bars = 50 μm.

Representative en face multiphoton images of the medial and adventitial layers of the ASC from WT and MU, male and female mice are shown in Fig. 2. The corresponding images for the DSC, SAB, and IAB are provided in Supp. Figs. S5–S7. Elastic fiber porosity, collagen fiber amounts, tortuosity, and thickness, and cell nuclei number and aspect ratio were quantified from the appropriate channels and layers.

#### 3.3.1. Elastic fiber porosity

**Fig. 3.**
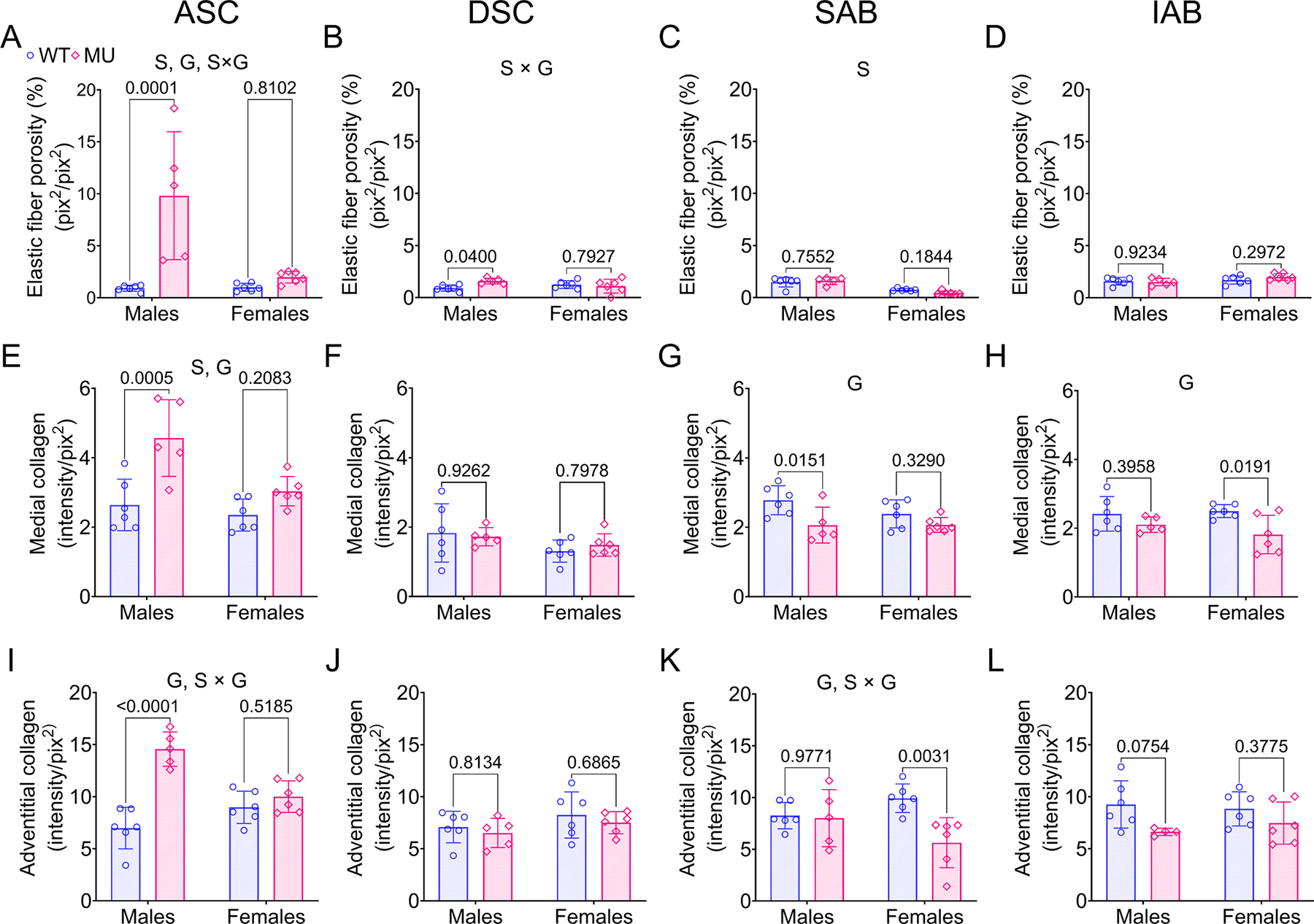
Medial elastic fiber porosity (A-D), medial collagen fiber content (E-H), and adventitial collagen fiber content (I-L) of ASC, DSC, SAB and IAB for each group. Methods are shown in Supp. Fig. S1. Letters indicate significant effects by two-way ANOVA for independent variables (sex (S) and genotype (G)). *P* values indicate significant difference between genotype for each sex by Tukey’s post hoc test. Individual data points and means ± SD are shown. *N* = 5 – 6/group. WT = blue circles, MU = pink diamonds.

Medial elastic fiber porosity for ASC, DSC, SAB, and IAB for WT and MU, male and female mice is shown in Fig. 3A–D. Elastic fiber porosity of the ASC was significantly higher in MU male mice compared to WT, whereas no significant difference was observed between MU and WT female mice (Fig. 3A). Sex, genotype, and interaction between sex and genotype contributed significantly to the variation in ASC elastic fiber porosity accounting for 19 % (*p* = 0.0042), 31 % (*p* = 0.0006), and 20 % (*p* = 0.0035), respectively. Elastic fiber porosity of DSC was also significantly higher in MU male mice than WT, with no significant differences between MU and WT female mice (Fig. 3B). There was a significant interaction between sex and genotype (*p* = 0.03581) accounting for 20 % of the total variation in the DSC. Elastic fiber porosity of SAB was similar between WT and MU mice, but was reduced in females compared to males (70 % variation, *p* < 0.0001) (Fig. 3C). Elastic fiber porosity in the IAB was similar across groups (Fig. 3D). These results show sex- and region-specific alterations of elastic fiber porosity in WT and MU aorta.

#### 3.3.2. Collagen content and fiber geometry

Collagen content in the medial and adventitial layers of ASC, DSC, SAB and IAB for MU and WT, male and female mice are shown in Fig. 3E–L, respectively. The medial and adventitial layers of ASC in MU male mice showed a significant increase in collagen content compared to WT (Fig. 3E, I). The collagen content in the medial layer of ASC was sex and genotype dependent accounting for 19 % (*p* = 0.0063) and 39 % (*p* = 0.0003) variation, respectively (Fig. 3E). The collagen content in the adventitial layer of ASC was significantly affected by genotype and the interaction between sex and genotype accounting for 48 % (*p* < 0.0001) and 28 % (*p* = 0.0002) variation, respectively (Fig. 3I). The collagen content in the medial and adventitial layers of DSC was similar between groups (Fig. 3F, J). Interestingly, the collagen content in the medial and adventitial layers of SAB were decreased in MU mice compared to WT with a significant dependence on genotype (30 % variation, *p* = 0.0056 in medial and 22 % variation, *p* = 0.015 in adventitial) (Fig. 3G, K). The collagen content in the adventitial layer of SAB was significantly affected by the interaction between sex and genotype with 18 % variation (*p* = 0.0293) (Fig. 3K). The collagen content in the medial layer of IAB was also reduced in MU compared to WT with a significant dependence on genotype (29 % variation, *p* = 0.0090), but was unchanged in the adventitial layer of the IAB (Fig. 3H, L).

**Fig. 4.**
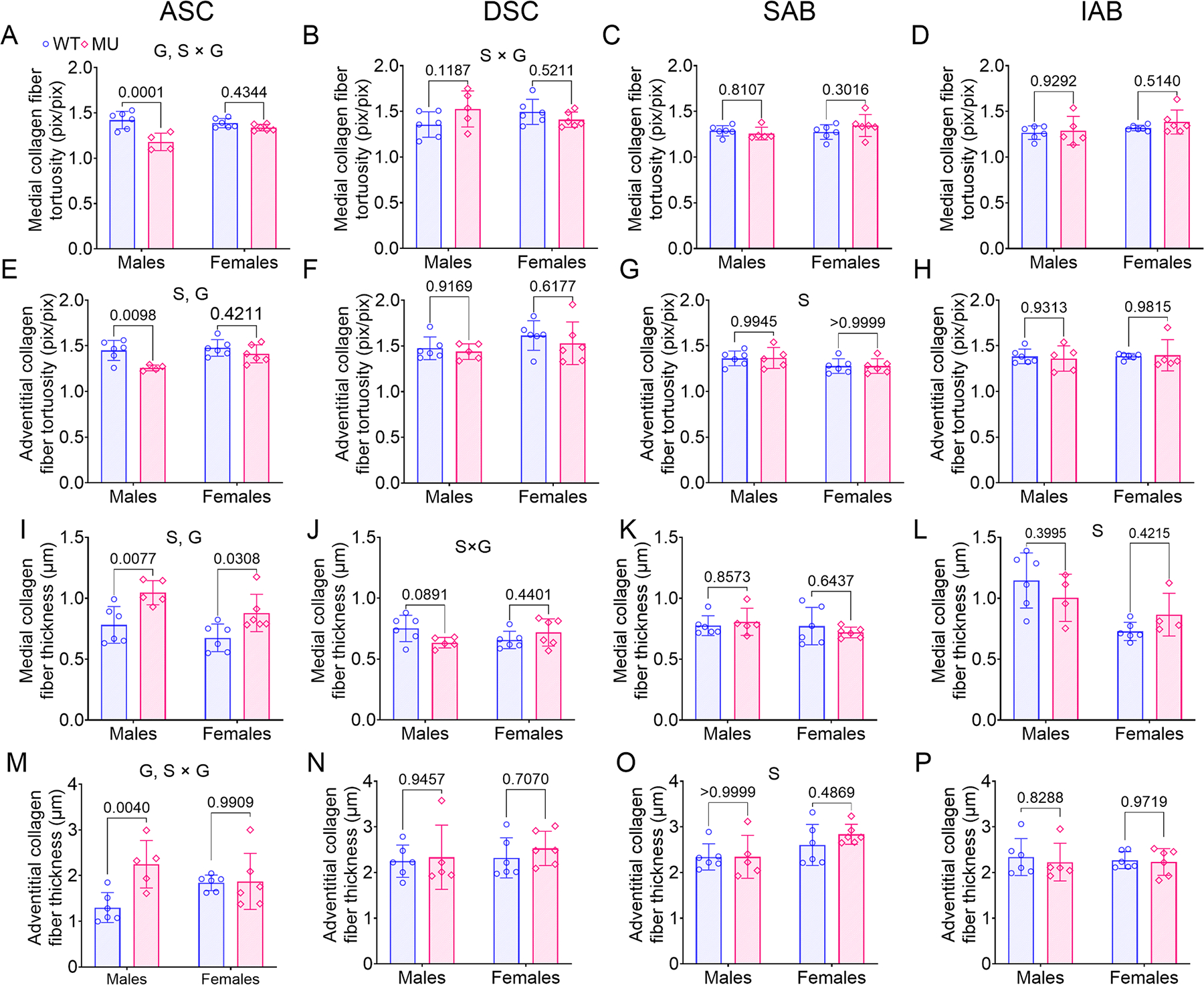
Collagen fiber tortuosity in (A-D) medial and (E-H) adventitial layers and collagen fiber thickness in (I-L) medial and (M-P) adventitial layers of ASC, DSC, SAB and IAB for each group. Methods are shown in Supp. Fig. S1. Letters indicate significant effects by two-way ANOVA for independent variables (sex (S) and genotype (G)). *P* values indicate significant difference between genotype for each sex by Tukey’s post hoc test. Individual data points and means ± SD are shown. *N* = 5 – 6/group. WT = blue circles, MU = pink diamonds.

To further delve into changes in collagen fiber organization, we determined the collagen fiber tortuosity and thickness in the medial and adventitial layers of ASC, DSC, SAB and IAB for MU and WT, male and female mice (Fig. 4). The collagen fiber tortuosity in the medial and adventitial layers of ASC was significantly lower in MU male mice compared to WT (Fig. 4A, E). The variation of collagen fiber tortuosity due to genotype for the medial and adventitial layers of ASC was 45 % (*p* = 0.0002) and 31 % (*p* = 0.005), respectively. There was a significant interaction between sex and genotype for medial collagen fiber tortuosity of ASC (Fig. 4A) and DSC (Fig. 4B) with 20 % (*p* = 0.0067) and 19 % (*p* = 0.0435) variation, respectively. The tortuosity of adventitial collagen fibers in ASC (Fig. 4E) and SAB (Fig. 4G) was significantly affected by sex with 16 % (*p* = 0.0359) and 23 % (*p* = 0.0247) variation. Genotype alone had no significant effect on collagen fiber tortuosity in any segment but the ASC (Fig. 4A – H).

Collagen fiber thickness in the medial and adventitial layers of the ASC, DSC, SAB, and IAB is shown in Fig. 4I–P. The collagen fibers in the medial and adventitial layers were significantly thicker in MU male ASC compared to WT (Fig. 4I, M). The thickness of medial collagen fibers in the ASC was significantly influenced by sex and genotype, accounting for 15 % (*p* = 0.0275) and 42 % (*p* = 0.0005) of the variation, respectively (Fig. 4I). The thickness of adventitial collagen fibers in the ASC showed a significant genotype effect, contributing to 22 % of the variation (*p* = 0.0151) and a significant interaction between sex and genotype, contributing 19 % of the variation (*p* = 0.0215) (Fig. 4M), respectively. Additionally, the thickness of medial collagen fibers in the IAB (Fig. 4L) and thickness of adventitial collagen fibers in SAB (Fig. 4O) was sex-dependent, with a variation of 35 % (*p* = 0.0023) and 23 % (*p* = 0.0231), respectively. Genotype alone had no significant effect on collagen fiber thickness in any segment but the ASC.

Overall, the MU ASC had increased collagen amounts, decreased collagen fiber tortuosity, and increased collagen fiber thickness that was exacerbated in males compared to females.

#### 3.3.3. Cell nuclei number and aspect ratio

**Fig. 5.**
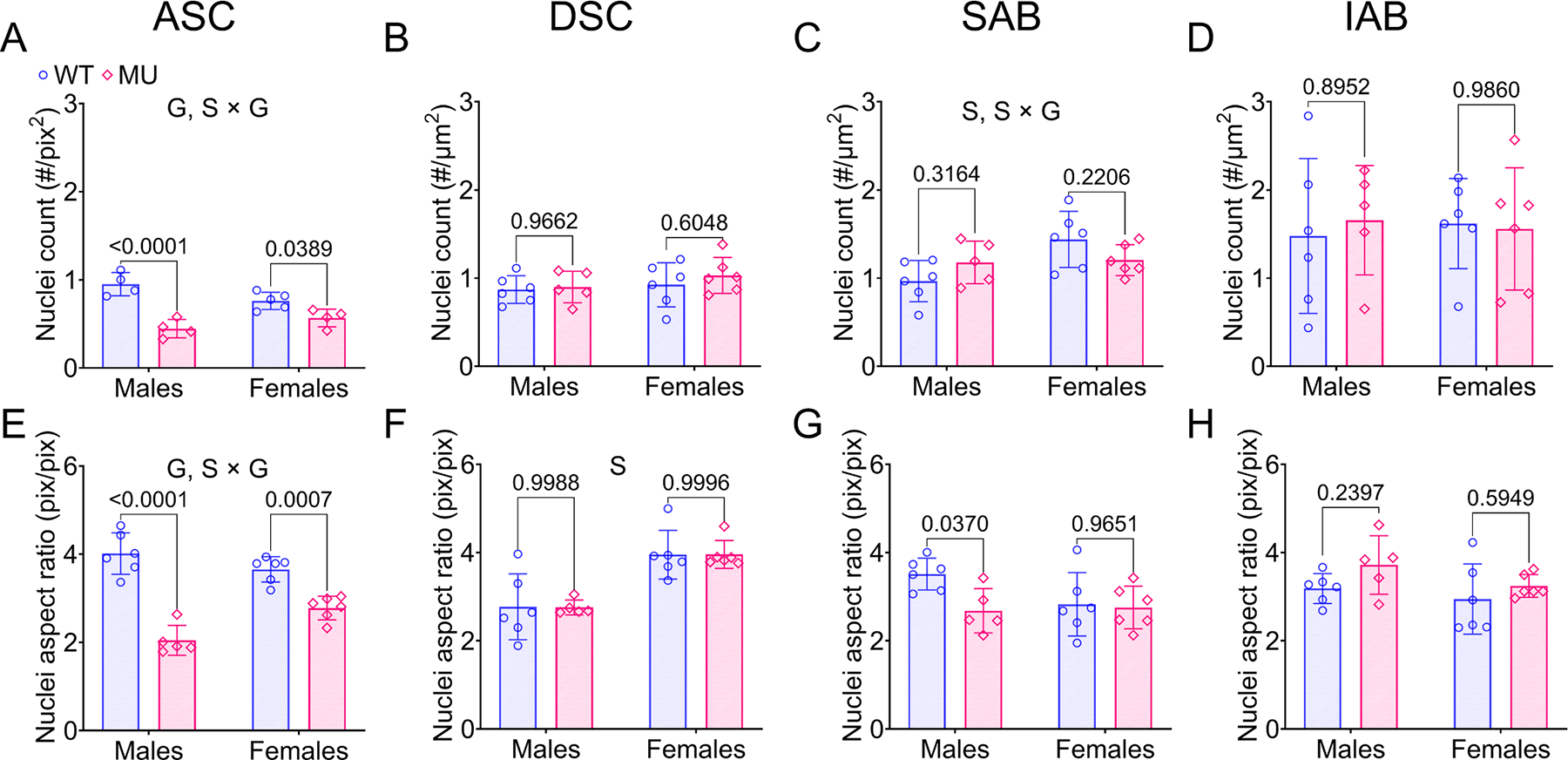
Cell nuclei count (A-D) and nuclei aspect ratio (E-H) in medial layer of ASC, DSC, SAB and IAB for each group. Methods are shown in Supp. Fig. S1. Letters indicate significant effects by two-way ANOVA for independent variables (sex (S) and genotype (G)). *P* values indicate significant difference between genotype for each sex by Tukey’s post hoc test. Individual data points and means ± SD are shown. *N* = 5 – 6/group. WT = blue circles, MU = pink diamonds.

Cell nuclei number and aspect ratio in the medial layer of ASC, DSC, SAB and IAB are shown in Fig. 5. Nuclei count in ASC was significantly lower in MU male and female mice than WT. The variation of nuclei count attributed to genotype was 69 % (*p* < 0.0001) and there was a significant interaction between sex and genotype contributing to 14 % of the variation (*p* = 0.0117) (Fig. 5A). The nuclei count in DSC and IAB did not show significant differences between MU and WT, male and female mice (Fig. 5B, D). For SAB, sex was a significant factor, explaining 20 % of the variation in nuclei count (*p* = 0.0235) and there was a significant interaction between sex and genotype, accounting for 16 % of the variation (*p* = 0.0440).

Nuclei aspect ratio = 1 indicates a perfect circle, while aspect ratio > 1 indicates increasingly ellipsoidal shape. The nuclei aspect ratio was significantly lower in male and female MU ASC compared to WT (Fig. 5E), indicating a more circular nuclei shape. Genotype was a significant factor in the nuclei aspect ratio and explained 75 % of the variability (*p* < 0.0001). An interaction between sex and genotype was also a significant factor contributing 12 % of the variation (*p* = 0.0104). Nuclei aspect ratio in DSC, SAB, and IAB did not depend on genotype (Fig. 5F–H). In DSC, sex had a significant effect on the aspect ratio, contributing to 62 % of the variability (*p* < 0.0001) (Fig. 5F).

The measures of cell nuclei number and aspect ratio are consistent with the elastic and collagen fiber results demonstrating sex- and region-specific wall remodeling in the MU ASC.

### 3.4. Passive mechanical behavior

**Fig. 6.**
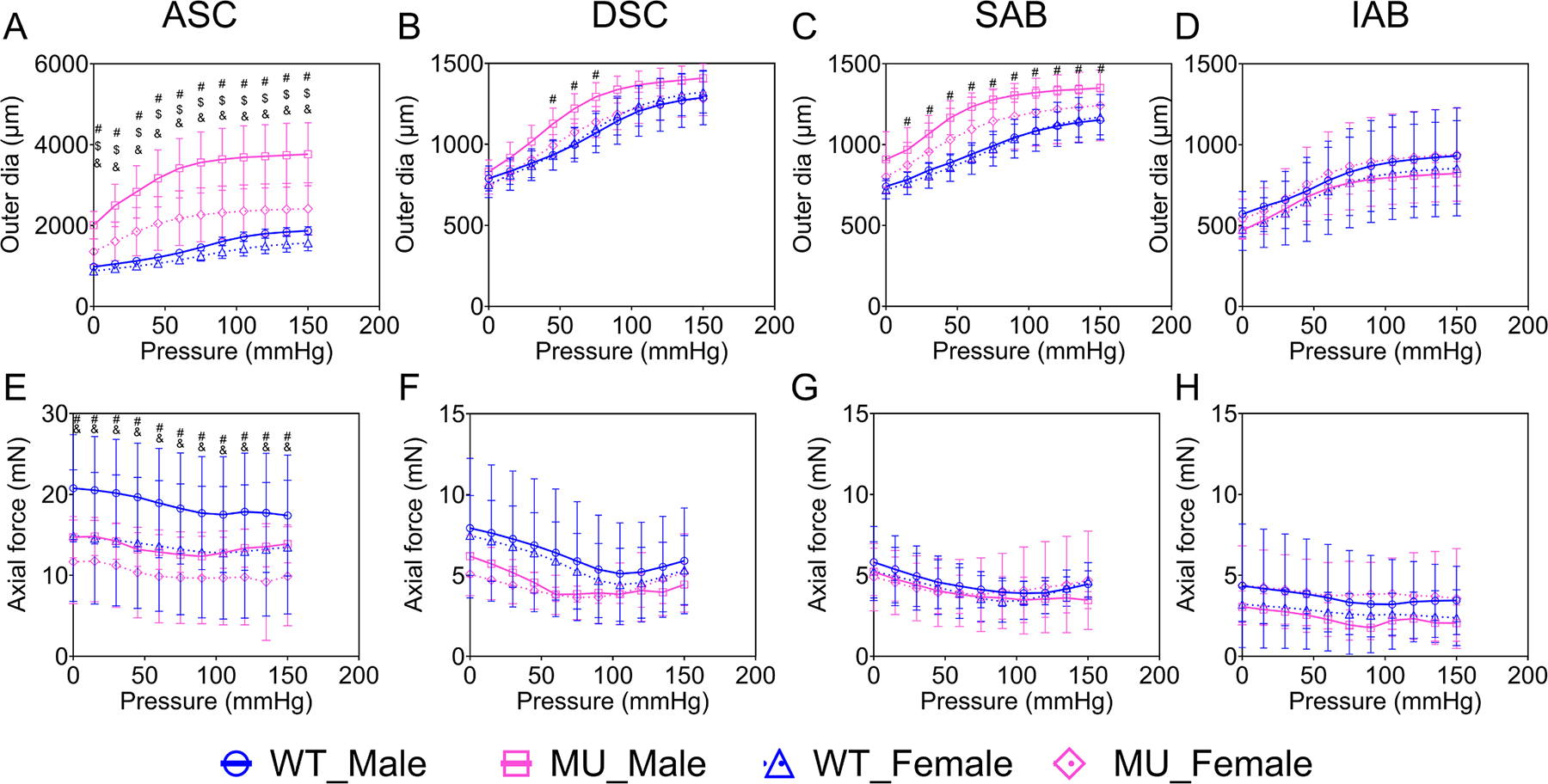
Pressure-outer diameter curves (mean ± SD) of ASC (A), DSC (B), SAB (C) and IAB (D) for each group. Pressure-axial force curves (mean ± SD) of ASC (E), DSC (F), SAB (G) and IAB (H) for each group. Note that the y-axes scales for A and E are larger than the others. *N* = 5 – 12/group. Comparisons between groups were made using two-way ANOVA with Tukey’s posthoc and p values < 0.05 are noted by # (WT males versus MU males), $ (WT females versus MU females) and & (MU males versus MU females). WT male = blue circles, MU male = pink squares. WT Female = blue triangles, MU Female = pink diamonds.

Pressure-outer diameter curves for the ASC, DSC, SAB, and IAB in male and female, WT and MU mice are shown in Fig. 6A–D. The outer diameter of ASC was significantly larger in MU male and female mice compared to WT at all pressure levels, with larger differences in males compared to females (Fig. 6A). Also, the outer diameter of ASC was significantly larger in males than females for both genotypes at all pressure levels. The outer diameter of DSC was significantly larger in MU male than WT male mice at pressures from 60 to 90 mmHg (Fig. 6B). The outer diameter of SAB was significantly larger in MU male than WT male mice at all pressure levels above 0 mmHg (Fig. 6C). The outer diameter of IAB was similar between groups for all pressures (Fig. 6D). These results show two important sex- and region-specific differences: 1) Both male and female MU ASC have significant dilation at all pressures, with larger dilation in males and 2) Only male MU mice show significant dilation in other aortic regions (DSC and SAB) and they only dilate in specific pressure ranges. Pressure-axial force curves for the ASC, DSC, SAB and IAB in male and female, WT and MU mice are shown in Fig. 6E–H. The minimal change in force as the aortic segment was inflated from 0 to 150 mmHg is indicative that it was at the physiologic axial stretch ratio [43]. For ASC, the axial force was significantly lower in MU male mice compared to WT male mice and in MU female mice compared to MU male mice (Fig. 6E). For other aortic segments, there were no significant differences in axial force between groups. Changes in axial force with genotype or sex in the ASC may be related to alterations in the physiologic axial stretch ratio or axial stiffness.

#### 3.4.1. Physiologic inner diameter

**Fig. 7.**
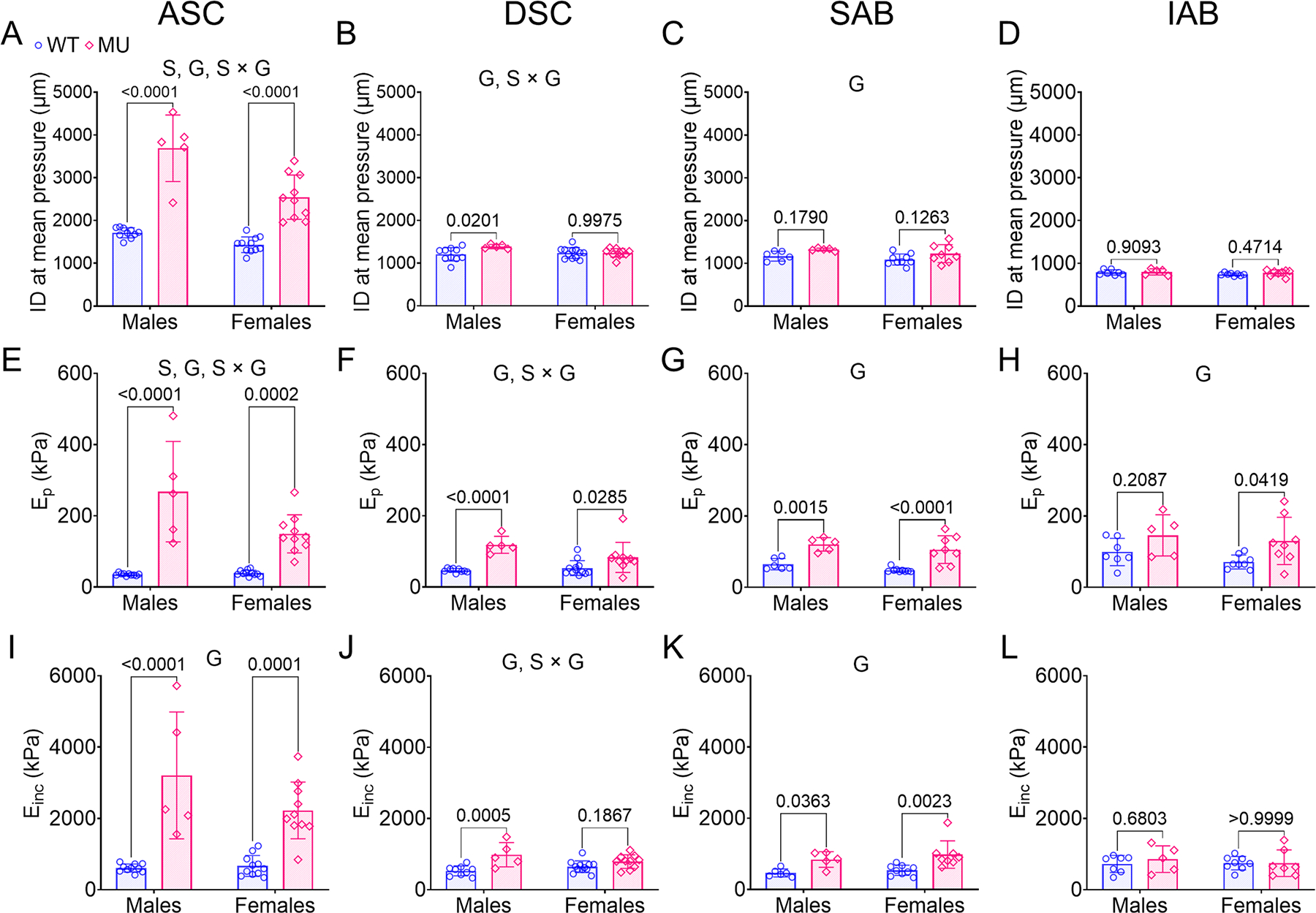
Inner diameter (ID) at mean pressure (A – D), physiologic circumferential structural stiffness (*E*_*p*_; E-H), and physiologic circumferential material stiffness (*E*_*inc*_; I-L) of ASC, DSC, SAB and IAB for each group. Letters indicate significant effects by two-way ANOVA for independent variables (sex (S) and genotype (G)). *P* values indicate significant difference between genotype for each sex by Tukey’s post hoc test. Individual data points and means ± SD are shown. *N* = 5 – 12/group. WT = blue circles, MU = pink diamonds.

The loaded inner diameter at mean blood pressure was calculated for each aortic segment and compared between groups (Fig. 7A–D). The inner diameter of ASC at mean pressure was significantly increased in MU mice compare to WT and was higher in male mice than female (Fig. 7A). There was a significant interaction between sex and genotype. The percent variation attributed to sex, genotype, and their interaction for inner diameter at mean pressure was 16 % (*p* < 0.0001), 74 % (*p* < 0.0001) and 6 % (*p* = 0.0048), respectively. For DSC, the inner diameter at mean pressure was significantly higher in MU male mice than WT, but was not significantly different between MU and WT female mice (Fig. 7B). The inner diameter of DSC at mean pressure was significantly affected by genotype (12 % variation, *p* = 0.0358) and there was a significant interaction between sex and genotype for the DSC inner diameter at mean pressure (11 % variation, *p* = 0.0428), indicating that the DSC, in addition to the ASC, shows sex dependent aortic dilation in this mouse model of MFS. For SAB (Fig. 7C) and IAB (Fig. 7D), the inner diameter at mean pressure was similar between MU and WT, male and female mice, although the SAB depended significantly on genotype (20 % variation, *p* = 0.0168). Overall, dilation in MU compared to WT mice was present in multiple aortic segments and increased in severity in proximal compared to distal regions and in males compared to females.

#### 3.4.2. Physiologic circumferential stiffnesses

*E*_p_ and *E*_inc_ are measures of the physiologic circumferential structural and material stiffness of the aortic wall, respectively. *E*_*p*_ for ASC, DSC, SAB and IAB for WT and MU, male and female mice is shown in Fig. 7E–H. *E*_*p*_ of ASC was significantly increased in MU male and female mice compared to WT and the differences were more pronounced in males than females (Fig. 7E). The significant contributions of sex, genotype, and their interaction in the variation of *E*_*p*_ for ASC was 8 % (*p* = 0.0076), 68 % (*p* < 0.0001), and 9 % (*p* = 0.0047), respectively. *E*_*p*_ of DSC was increased significantly in MU male and female mice compared to WT (Fig. 7F). There were significant effects of genotype (47 % variation, *p* < 0.0001) and the interaction between sex and genotype (8 % variation, *p* = 0.0352) for the DSC. *E*_*p*_ of SAB and IAB was increased significantly in MU male and female mice compared to WT (Fig. 7G, H), respectively. Genotype accounted for 57 % (*p* < 0.0001) and 24 % (*p* = 0.0086) of the variation in *E*_*p*_ for the SAB and IAB, respectively.

*E*_*inc*_ for ASC, DSC, SAB and IAB for MU and WT, male and female mice is shown in Fig. 7I–L. *E*_*inc*_ of ASC was significantly higher in MU male and female mice compared to WT, with larger differences in males than females (Fig. 7I). Variations in ASC *E*_*inc*_ were significantly attributed to genotype (65 %, *p* < 0.0001). For DSC, *E*_*inc*_ was significantly higher in MU male mice compared to WT (Fig. 7J). For DSC, *E*_*inc*_ variations were significantly attributed to genotype (36 %, *p* = 0.0001), and interaction between sex and genotype (10 %, *p* = 0.0325). For SAB, *E*_*inc*_ was greater in MU male and female mice compared to WT (Fig. 7K), consistent with the *E*_*p*_ data. For SAB, genotype accounted for 40 % of *E*_*inc*_ variation (*p* = 0.0003). *E*_*inc*_ of IAB was similar across genotypes and sexes (Fig. 7L).

The physiologic circumferential stiffness values show increases in structural and material stiffnesses in MU compared to WT mice in almost all aortic regions, with more pronounced differences in proximal compared to distal regions and in males compared to females.

#### 3.4.3. Physiologic stretches and stresses

**Fig. 8.**
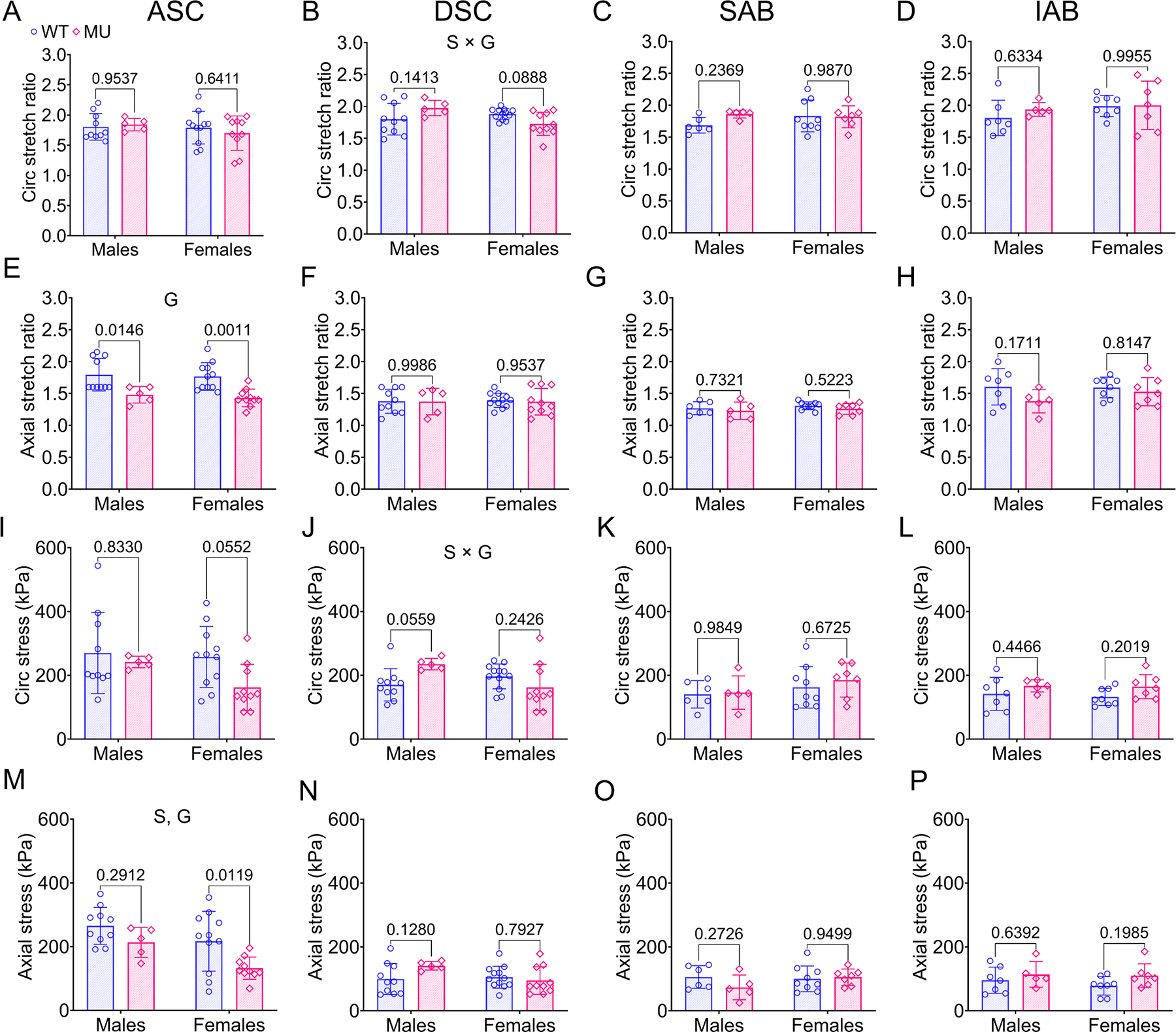
Physiologic circumferential stretch ratio (A-D), axial stretch ratio (E-H), circumferential stress (I-L), and axial stress (M-P) of ASC, DSC, SAB and IAB for each group. Letters indicate significant effects by two-way ANOVA for independent variables (sex (S) and genotype (G)). *P* values indicate significant difference between genotype for each sex by Tukey’s post hoc test. Individual data points and means ± SD are shown. *N* = 5 – 12/group. WT = blue circles, MU = pink diamonds.

The physiologic circumferential stretch ratio at mean blood pressure across all aortic segments was similar between MU and WT, male and female mice (Fig. 8A–D). In the DSC, circumferential stretch showed a significant interaction between sex and genotype (18 % variation, *p* = 0.0099). The physiologic axial stretch ratio of the ASC was significantly less in MU male and female mice compared to WT (Fig. 8E) and the variation in axial stretch ratio was significantly attributed to genotype (38 %, *p* < 0.0001). The decrease in axial stretch of the male MU ASC compared to the male WT ASC was consistent with the lower axial force values (Fig. 6E). For other aortic segments, the physiologic axial stretch was similar between MU and WT, male and female mice (Fig. 8F–H). This indicates a region-specific effect on axial remodeling of the MU aorta.

Like the circumferential stretch results, circumferential stress was similar between MU and WT, male and female mice across all aortic segments (Fig. 8I–L). In the DSC, circumferential stress showed a significant interaction between sex and genotype (19 % variation, *p* = 0.0093). In the ASC, axial stress was significantly decreased in MU compared to WT (genotype = 17 % variation, *p* = 0.0056) and in females compared to males (sex = 15 % variation, *p* = 0.0084) (Fig. 8M). The decrease in axial stress for the ASC was consistent with the decrease in axial force (Fig. 6E). Axial stress was similar between groups for the DSC, SAB and IAB (Fig. 8N–P). The physiologic stretches and stresses show that overall MU aorta can adapt to maintain WT levels of stretch and stress, likely through remodeling of the unloaded dimensions (Fig. 1). MU ASC shows additional axial adaptation of the axial stretch and resulting axial stress that may be in response to the large increases in circumferential stiffness (Fig. 7E, I) as unloading in the axial direction can decrease stress and stiffness in the circumferential direction due to coupling between directions [49]. MU ASC may also have altered axial stiffness (not measured here) that would affect axial stress.

#### 3.4.4. Ex vivo circumferential material stiffnesses

**Fig. 9.**
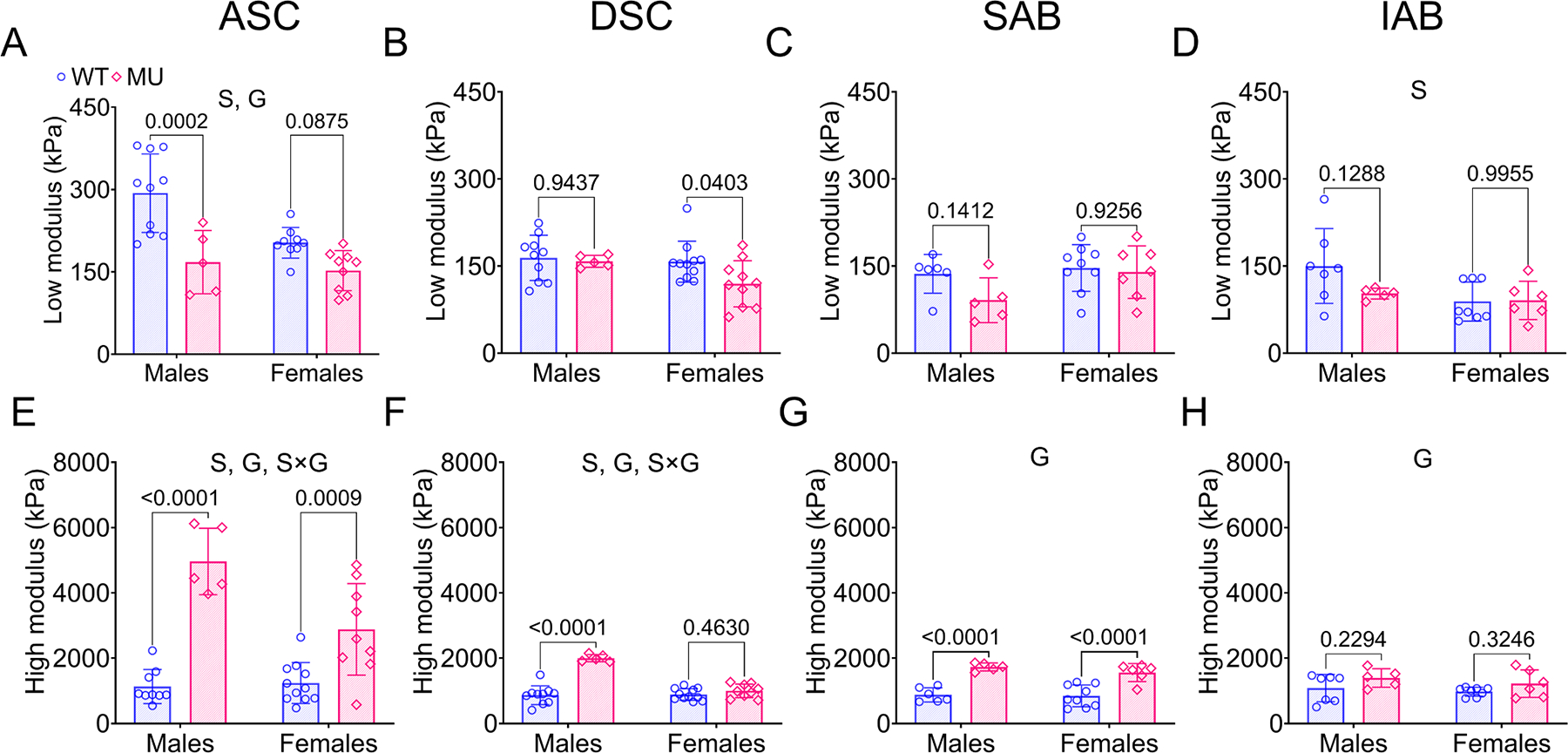
Ex vivo circumferential material stiffness as measured by the low (A-D) and high moduli (E-H) for ASC, DSC, SAB, and IAB for each group. Methods for calculating the moduli are shown in Supp. Fig. S3. Letters indicate significant effects by two-way ANOVA for independent variables (sex (S) and genotype (G)). *P* values indicate significant difference between genotype for each sex by Tukey’s post hoc test. Individual data points and means ± SD are shown. *N* = 5 – 11/group. WT = blue circles, MU = pink diamonds.

The low and high moduli, ex vivo circumferential material stiffness of the aortic wall under different loading conditions, are indicators of mechanical contributions of the elastic and collagen fibers, respectively [46,47]. The low and high moduli were obtained from linear fits to the circumferential stretch-stress curves (Supp. Fig. S3). The average stretch-stress curves for each aortic segment are provided in Supp. Fig. S8. The low modulus was reduced in MU male mice compared to WT for ASC, with sex and genotype accounting for 12 % (*p* = 0.0079) and 32 % (*p* < 0.0001) of the variability, respectively (Fig. 9A). There were no significant differences due to genotype in the low modulus for other aortic segments (Fig. 9B–D). The low modulus was reduced in females compared to males in the IAB (sex = 15 % variation, *p* = 0.0399).

The high modulus for each aortic segment for WT and MU, male and female mice is shown in Fig. 9E–H. The high modulus was increased in MU males compared to WT males in ASC, DSC, and SAB and increased in MU females compared to WT females in ASC and SAB (Fig. 9E–G). The high modulus depends on sex, genotype, and interactions between sex and genotype in the ASC (sex = 9 % variation, *p* = 0.0060; genotype = 66 % variation, *p* < 0.0001; sex × genotype = 11 % variation, *p* = 0.0026) and DSC (sex = 29 % variation, genotype = 47 % variation, *p* < 0.0001, *p* < 0.0001; and sex × genotype = 32 % variation, *p* < 0.0001) (Fig. 9E and F). The high modulus depends on genotype only in the SAB (variation = 69 %, *p* < 0.0001) (Fig. 9G) and IAB (variation = 16 %, *p* = 0.0451) (Fig. 9H).

The results for the low and high moduli show that the circumferential mechanical contributions attributed to elastic and collagen fibers vary in a sex- and region-dependent fashion in MU mice. The elastic fiber contributions (low modulus) are decreased only the aneurysm-susceptible ASC, while the collagen fiber contributions (high modulus) are increased in all aortic segments with more significant increases in males compared to females.

### 3.5. Correlation analyses

#### 3.5.1. Individual correlations

Relationships between various metrics including microstructural remodeling, mechanical metrics, and unloaded dimensions for each individual aortic segment and pooled data are illustrated using a Pearson correlation matrix (Supp. Figs. S9–S13) with corresponding *p*-values (Supp. Tables S2–S6). As we were primarily interested in how microstructural remodeling and mechanical metrics may influence aneurysm progression (i.e. aortic dilation), we further investigated correlations between each metric and the inner diameter at mean blood pressure.

**Fig. 10.**
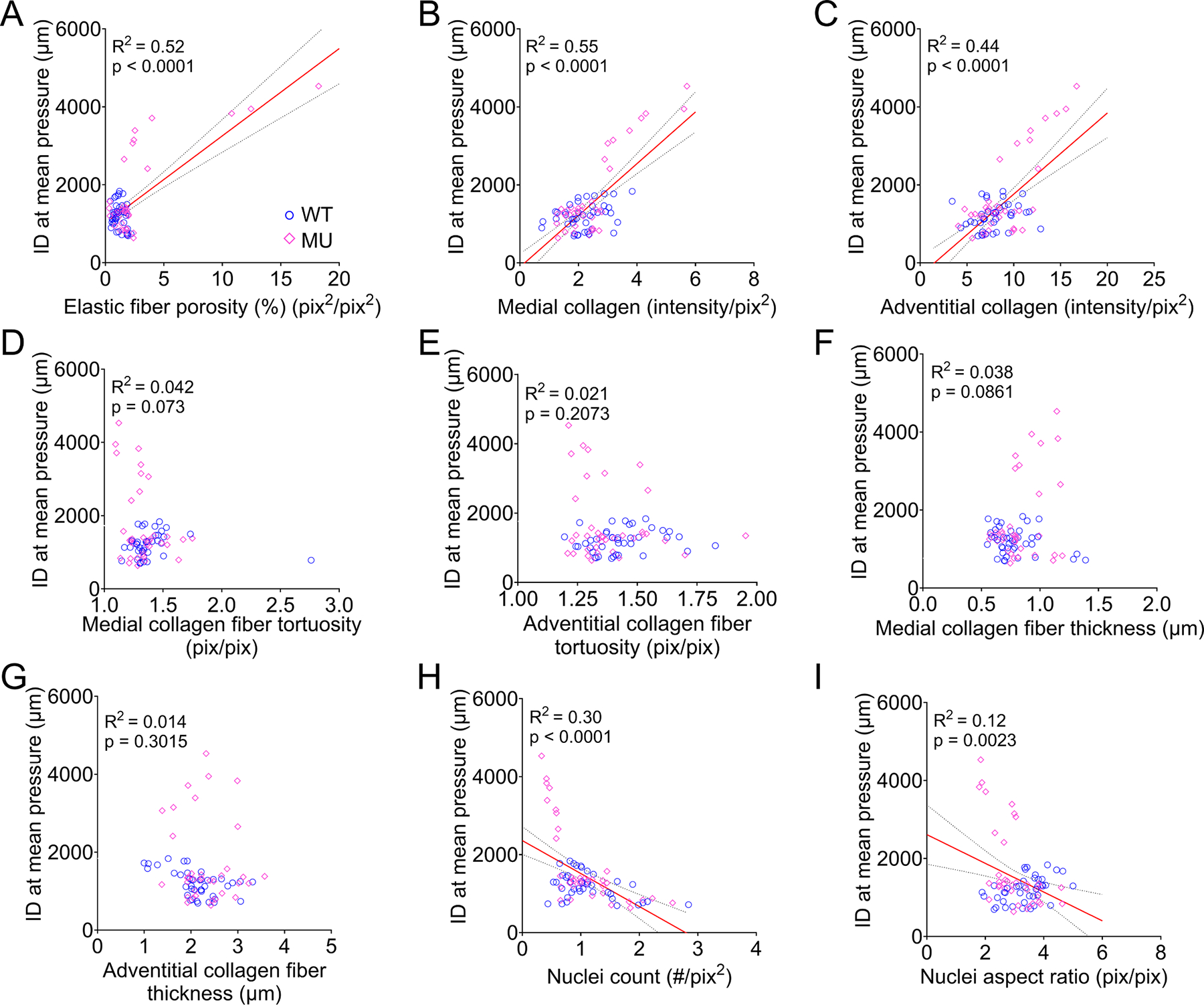
Pearson correlation with linear regression analysis between inner diameter at mean pressure (an indicator of aortic dilation) and microstructural remodeling metrics. Data for ASC, DSC, SAB, and IAB of WT and MU, male and female mice were pooled for correlation analysis. Correlation plots between inner diameter at mean pressure and elastic fiber porosity (A), medial collagen content (B), adventitial collagen content (C), medial collagen fiber tortuosity (D) and adventitial collagen fiber tortuosity (E),), medial collagen fiber thickness (F), adventitial collagen fiber thickness (G), nuclei count (H), nuclei aspect ratio (I). R^2^ and *P* value in each panel indicates goodness of fit and significance, respectively. WT = blue circles, MU = pink diamonds.

Fig. 10 presents correlation relationships between the inner diameter at mean pressure and microstructural remodeling metrics. The inner diameter showed significant positive correlations with elastic fiber porosity (Fig. 10A) and medial (Fig. 10B) and adventitial (Fig. 10C) collagen fiber content, and significant negative correlations with nuclei count (Fig. 10H) and nuclei aspect ratio (Fig. 10I). There were no significant correlations between inner diameter at mean pressure and medial (Fig. 10D, F) and adventitial (Fig. 10E, G) collagen fiber tortuosity and thickness. Among the microstructural remodeling metrics, medial collagen fiber content (R^2^ = 0.55) (Fig. 10B) and elastic fiber porosity (R^2^ = 0.52) (Fig. 10A) were the strongest individual predictors of aortic dilation.

**Fig. 11.**
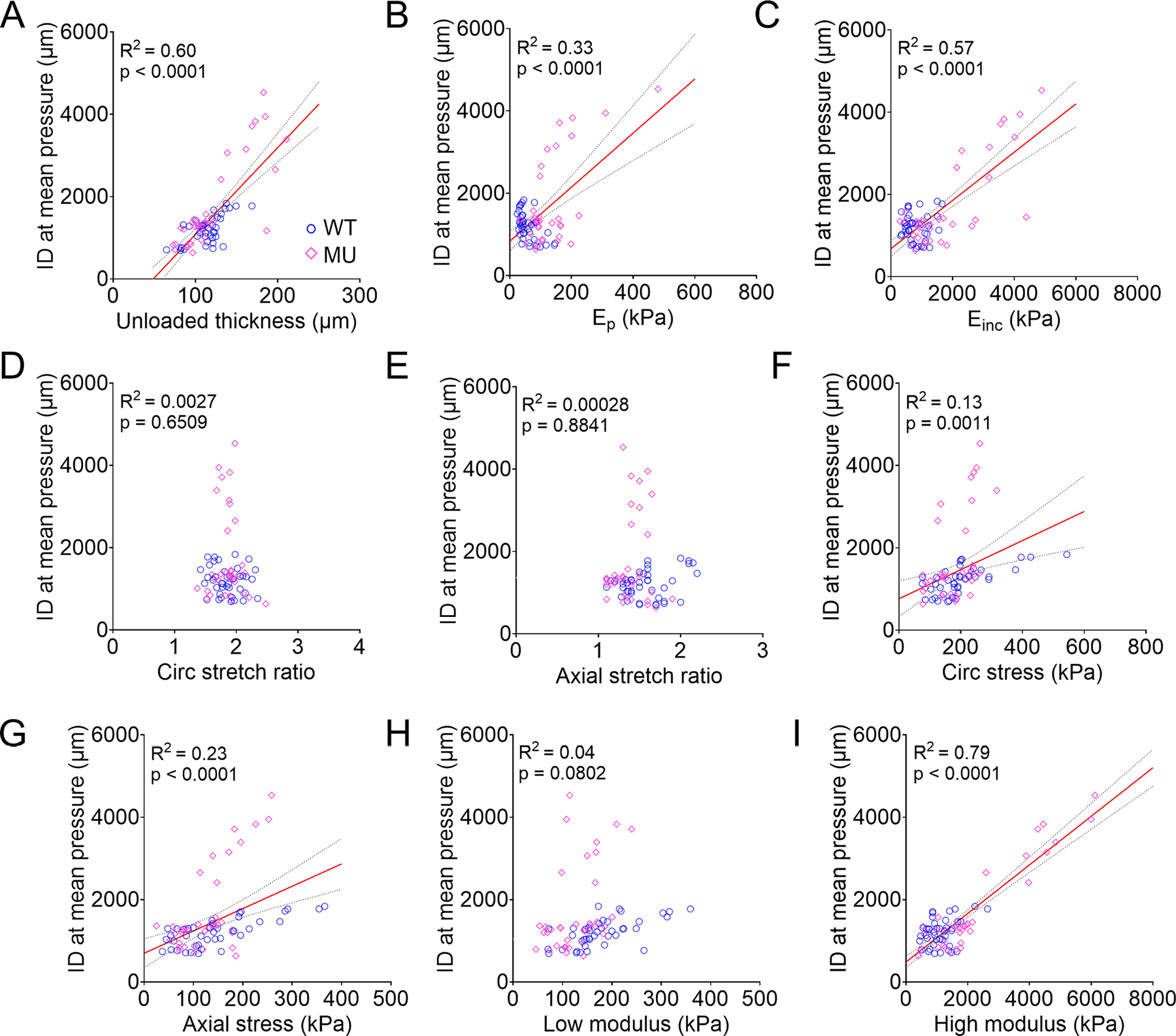
Pearson correlation with linear regression analysis between inner diameter at mean pressure (an indicator of aortic dilation) and wall thickness and mechanics metrics. Data for ASC, DSC, SAB and IAB aorta of WT and MU, male and female mice were pooled for correlation analysis. Correlation plots between inner diameter at mean pressure and unloaded thickness (A), physiologic circumferential structural stiffness (*E*_*p*_) (B), physiologic circumferential material stiffness (*E*_*inc*_) (C), circumferential stretch ratio (D), axial stretch ratio (E), circumferential stress (F), axial stress (G), low modulus (H), and high modulus (I). R^2^ and *P* value in each panel indicate goodness of fit and significance, respectively. WT = blue circles, MU = pink diamonds.

Fig. 11 presents correlation relationships between the inner diameter at mean pressure and the unloaded thickness and mechanical metrics. The inner diameter demonstrated a significant positive correlation with unloaded thickness (Fig. 11A), physiologic structural stiffness (*E*_*p*_) (Fig. 11B), physiologic material stiffness (*E*_*inc*_) (Fig. 11C), circumferential (Fig. 11F) and axial (Fig. 11G) stress, and high modulus (Fig. 11I). No significant correlation was observed between inner diameter and circumferential (Fig. 11D) or axial (Fig. 11E) stretches or low modulus (Fig. 11H). Among the unloaded dimensions and mechanical metrics, high modulus was the strongest predictor of aortic dilation (R^2^ = 0.79) (Fig. 11I).

#### 3.5.2. Multivariable mixed model analyses

Stepwise multivariable regression using a mixed model was conducted to determine the combined contribution of metrics and to construct the most effective predictive models for accurately assessing aortic dilation. For model development, three cases were evaluated: Case 1 involved inner diameter versus microstructural remodeling, Case 2 involved inner diameter versus mechanical metrics, and Case 3 involved inner diameter versus the combination of microstructural remodeling, mechanical metrics, and unloaded dimensions.

**Fig. 12.**
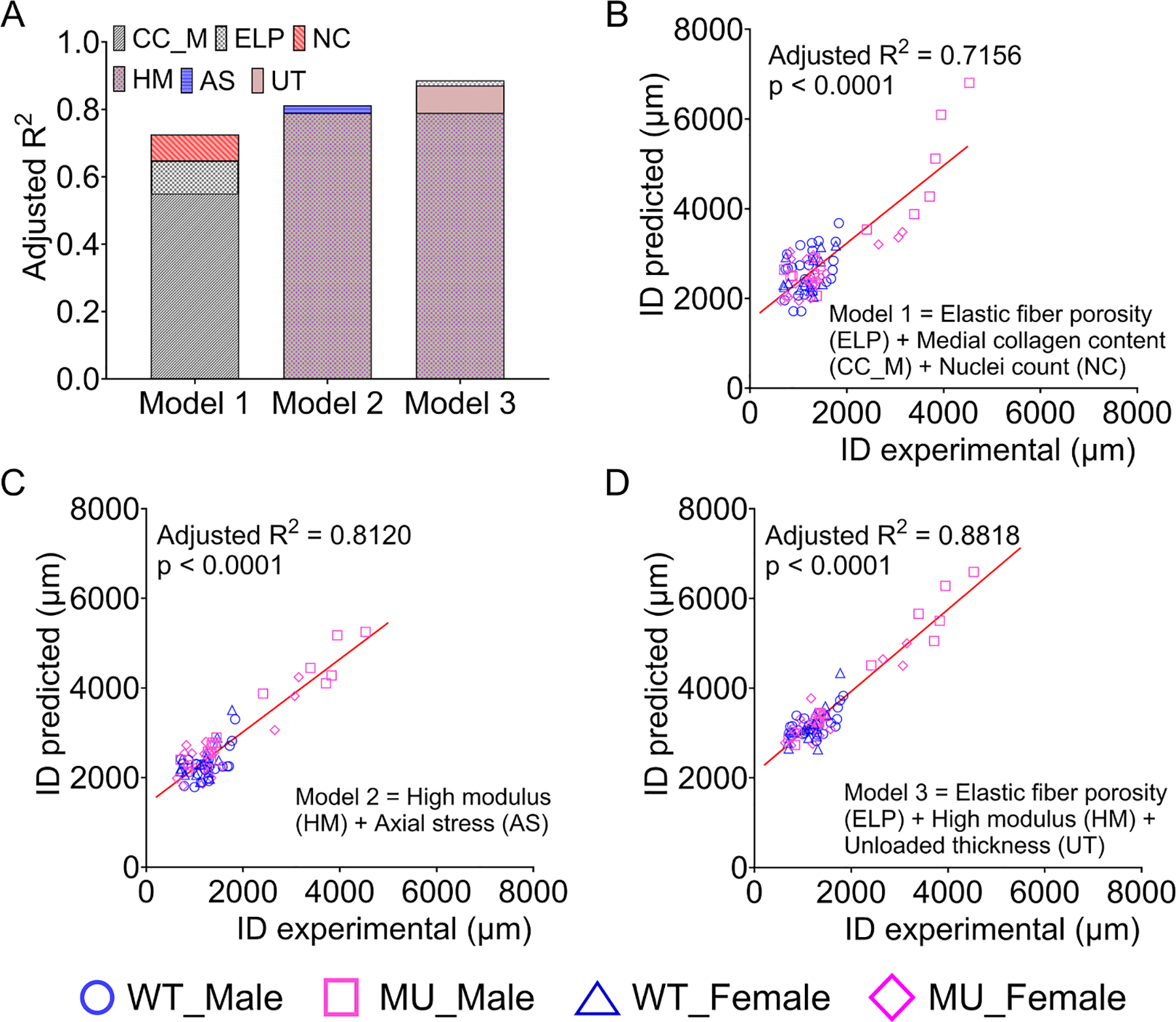
Stepwise multivariable regression was used to compare the adjusted R^2^ (goodness of fit) between the best models for each case (A). The predicted and experimental inner diameter (ID) at mean pressure were plotted for the best model for each case (B – D). Case 1 included only microstructural remodeling metrics. Model 1 showed that the combination of elastic fiber porosity (ELP), medial collagen content (CC_M), and nuclei count (NC) were the best predictors of aortic dilation for case 1 (B). Case 2 included only mechanical metrics. Model 2 showed that high modulus (HM) and axial stress (AS) were the best predictors of aortic dilation for case 2 (C). Case 3 included microstructural remodeling, mechanical metrics, and unloaded dimensions. Model 3 showed that elastic fiber porosity, high modulus, and unloaded thickness (UT) were the best predictors of aortic dilation for case 3 (D). Model 3 had the highest overall R^2^. WT male = blue circles, MU male = pink squares. WT Female = blue triangles, MU Female = pink diamonds.

In Case 1, where microstructural remodeling metrics were considered, the combination of elastic fiber porosity, medial collagen content, and nuclei count (Model 1) exhibited the highest predictive capability for inner diameter at mean pressure. In Model 1, medial collagen content emerged as the most influential contributor, as evidenced by its higher impact on the adjusted R^2^ value (Fig. 12A). The predictive performance of Model 1 is illustrated in Fig. 12B.

In Case 2, where mechanical metrics were considered, the combination of high modulus and axial stress (Model 2) demonstrated the highest predictive capacity for the inner diameter at mean pressure. In Model 2, the high modulus was identified as the primary contributor (Fig. 12A), indicating the dominant role of the high modulus in predicting aortic dilation. The predictive capacity of Model 2 is demonstrated in Fig. 12C. Between the two models, Model 2 (adjusted R^2^ = 0.8120) demonstrated greater predictive power compared to Model 1 (adjusted R^2^ = 0.7156). This suggests that mechanical metrics are better predictors of aortic dilation than microstructural remodeling metrics.

Case 3 included the combined effect of microstructural remodeling, mechanical metrics, and unloaded dimensions. Among these metrics, the combination of high modulus, elastic fiber porosity, and unloaded thickness (Model 3) demonstrated the highest predictive power. In Model 3, high modulus emerged as the dominant metric, contributing most significantly to the predictive power (Fig. 12A), reinforcing the important role of this ex vivo circumferential stiffness measure in predicting aortic dilation. Elastic fiber porosity and unloaded thickness had small, but significant roles in Model 3, demonstrating how multiple types of data (i.e. microstructural remodeling, mechanical metrics, and unloaded dimensions) are needed to improve predictive power. Note that wall thickness is a critical determinant of wall stress (Eqn. 3). Even though wall stresses were not strong predictors of aortic dilation (Fig. 11F, G), remodeling responses associated with increased wall stress (i.e. increased wall thickness) may play an important role. Among the three models, Model 3 exhibited the strongest overall predictive capacity for the inner diameter at mean pressure of the aorta with an adjusted R^2^ = 0.8818 (Fig. 12D).

The coefficients of independent variables, p-values, and variance inflation factors corresponding to each model are given in Table 2.

## 4. Discussion

We used a severe mouse model of MFS to investigate sex- and region-specific effects in the progression of aortic aneurysm. We collected data on unloaded dimensions, microstructural remodeling, and passive wall mechanics from four aortic regions (ASC, DSC, SAB, and IAB) of *Fbn1*^*mgR/mgR*^ (MU) and littermate WT male and female mice at 4 months of age and correlated them with aortic dilation. Our findings are consistent with previously reported studies investigating regional variations in aortic microstructural remodeling and wall mechanics in MFS mouse models. However, previous studies were limited to the thoracic aorta (ASC and DSC) [7,9,23,50] and usually male mice [9,23,50].

### 4.1. Unloaded wall dimensions and circumferential residual stretch

Increases in unloaded diameter of the aorta demonstrate permanent dilation or plastic circumferential deformation, while increases in wall thickness reflect addition of mass through ECM remodeling and cellular events [51,52]. We observed that unloaded geometry in MU aorta varies by region. Notably, there was significantly larger permanent dilation and increased wall thickness observed in MU ASC compared to WT that was not present in other regions (Fig. 1A–H). The permanent dilation of MU ASC could be the result of decreased wall elasticity or resilience due to a decrease of medial elastic fiber content [8], as evidenced by an increase in elastic fiber porosity compared to WT (Fig. 3A). Bellini et al. [23] and Cavinato et al. [9] also noted large increases in ASC dimensions compared to DSC, but they only examined male mice. Our results showed similar region-specific adaptation of the aortic dimensions in male and female MU ASC.

In healthy individuals, the diameter and thickness of the aortic wall adapt over time to maintain mechanical homeostasis under physiological loading [30]. Importantly, the ratio of diameter to thickness remains constant [30], ensuring constant physiological circumferential stress (Eqn. 3a) on the wall over the life span. The diameter to thickness ratio was unchanged in MU ASC compared to WT (Fig. 1I), indicating that wall remodeling was able to maintain circumferential wall stress despite aneurysm progression. Although genotype did affect the diameter to wall thickness ratio in some other aortic segments (Fig. 1J, L), circumferential stresses were maintained at similar levels in both genotypes for all aortic segments (Fig. 8I–L).

The circumferential residual stretch ratios are also indicators of wall remodeling [53]. Our findings of compressive residual stretch at the inner wall (Supp. Figs. S4A–D) and tensile residual stretch at the outer wall (Supp. Figs. S4E–H) with approximately constant values along the aortic length are consistent with previous results in pig [54], human [55], and mouse [56]. The dependence of residual stretch on genotype for the inner and outer radii for the ASC and for the inner radii for the DSC suggest wall remodeling in these segments for MU mice. Like the unloaded dimensions, changes in the residual stretch did not depend on sex, indicating similar remodeling in male and female MU aorta.

### 4.2. Remodeling of ECM and cells in the aortic wall

Geometric adaptation of the aortic wall is caused by synthesis, degradation, and remodeling of cells and ECM during aneurysm progression [8,57]. Male MU ASC exhibited the most pronounced changes in wall microstructure, including increased elastic fiber porosity (Fig. 3A), increased **c**ollagen amounts (Fig. 3E, I) and fiber remodeling (Fig. 4A, E, I, M), and loss of cell nuclei (Fig. 5A) and their elliptical shape (Fig. 5E). Cavinato et al. [9] and Chen et al. [50] demonstrated that decreases in elastic fiber porosity, increases in collagen deposition, and decreases in cell nuclei density were more severe in ASC than DSC in mouse models of MFS, but they only included male mice. Our results show that female MU ASC is partially protected from these microstructural changes compared to male MU ASC.

Loss of cell nuclei and changes in shape from elliptical to circular could be indicative of detachment of cells from fragmented elastic fibers [2] or apoptosis or phenotype switching under high local mechanical stresses [58]. Alterations in cell phenotype may promote abnormal ECM remodeling, such as deposition of disorganized collagen fibers or proteoglycan accumulation, that further contribute to aneurysm progression [59,60]. Human studies show similar differences in microstructural remodeling to those observed here, highlighting loss of elastic fibers and increased collagen in aneurysmal compared to non-aneurysmal regions [61,62].

The region-specific microstructural remodeling of the aorta may result from differences in embryonic lineage of cells [27,28] and/or physiological loading [32] across regions. These region-specific variations regulate the local bioavailability of growth factors and cytokines [27,28], SMC reactivity and phenotype switching [27], aortic contractility [63], as well as the expression of matrix metalloproteinases (MMP-2 and MMP-9) and their inhibitors (TIMPs) [64,65]. This region-specific microstructural remodeling directly influences the aortic wall biomechanics [7,23,66] through a positive feedback loop [4]. This interplay makes the aorta more susceptible to dilation and eventual rupture, particularly in regions already prone to mechanical and structural vulnerabilities, such as the ASC [4]. The sex-specific effects on microstructural remodeling in the MU aorta may stem from differences in sex hormones, as estrogen plays a protective role by reducing vascular inflammation, which slows aneurysm development and progression in women [21,67]. Additionally, higher levels of TIMPs in women [68] due to two homogametic chromosomes may contribute to reduced ECM degradation, serving as another protective mechanism. Human data on region- and sex-specific ECM and cell remodeling in MFS is limited and future studies are needed to support and strengthen the findings from animal research.

### 4.3. Aortic dilation and passive mechanical behavior

Dilation (increased inner diameter at mean pressure) was most pronounced in MU male ASC, followed by MU female ASC. ASC was the only aortic region with significant effects of sex on dilation. Significant genotype and/or sex and genotype interaction effects were noted in ASC, DSC and SAB, with no significant differences in dilation between groups for IAB (Fig. 7A–D). These results indicate that outcomes of MFS in terms of aneurysm development are more severe in the thoracic aorta, particularly in males. Other studies on mouse models of MFS have also demonstrated large dilation of the ASC, with smaller, but measurable dilation in the DSC for males [9,23].

Like the dilation, the increases in physiologic circumferential structural, *E*_*p*_, and material, *E*_*inc*_ stiffness, were the largest in male MU ASC, followed by MU female ASC. ASC was the only aortic region with significant effects of sex on *E*_*p*_. Significant genotype and/or sex and genotype interaction effects for *E*_*p*_ and *E*_*inc*_ were noted in ASC, DSC, SAB, and IAB (*E*_*p*_ only). (Fig. 7E–L). ASC was the only MU aortic region with changes in multiple microstructural remodeling metrics for elastic fibers, collagen, and SMCs that significantly depended on genotype, suggesting that these microstructural changes may be linked to the large increases in *E*_*p*_ and *E*_*inc*_. Previous studies also showed increased physiologic circumferential stiffness [9,23,50] and increased elastic fiber porosity, increased collagen fiber straightness, and decreased SMC density [9,50] of male ASC and DSC in mouse models of MFS. Since DSC, SAB, and IAB showed genotype dependent increases in *E*_*p*_ and *E*_*inc*_, but did not consistently show genotype dependent microstructural alterations like those observed in the ASC, there were likely additional ECM and SMC changes in those regions that could be measured in future work through genetic [69,70], biochemical [57,71], or imaging [60,72] techniques. Axial stiffness of the aortic regions was not measured in the current study. Although Bellini et al. [23] found no differences in axial stiffness between WT and MU ASC, Ferruzzi et al. [73] found differences in axial stiffness of different aortic segments and alterations with aging in mice that may be important for region-specific TAA progression.

The high and low moduli, additional circumferential stiffness measures, can be obtained through ex vivo experiments under varying loading conditions. These provide more specific measurements of aortic mechanical properties, since the aorta is isolated from surrounding tissues during testing and can be exposed to a large loading range [74]. However, partially equivalent in vivo measures could be obtained using drugs, physical activity, or natural diurnal variation to decrease or increase blood pressure [75]. While low moduli increases in MU mice were specific to ASC, genotype-dependent high moduli increases were observed in ASC, DSC, SAB, and IAB with larger changes in males than females (Fig. 9). High moduli showed the highest individual correlation with inner diameter at mean pressure of all the independent variables (Fig. 11I). Clinical studies have demonstrated a similar region-specific trend to that reported here for aortic dilation and circumferential structural stiffness when stiffness was measured by pulse wave velocity (PWV) in MFS patients, but results were not separated by sex [16,31]. Additional studies are needed to investigate sex differences in region-specific aortic dilation and passive mechanical behavior in humans with MFS.

### 4.4. Predictions of aortic dilation

Pearson correlation results showed that measures of circumferential material wall stiffness (*E*_*inc*_ and high modulus) were predictive of aortic aneurysm dilation (Fig. 11C, I). Both measures are related to the capacity of the aorta to deform and store strain energy during systole that is needed during diastole to reduce the work of the heart and dampen pressure pulse waves [2]. The increases in physiologic circumferential material stiffness (*E*_*inc*_) of the aortic wall in MU compared to WT varied from ~5.3x (male, ASC), ~3.3x (female, ASC) and ~ 1.9x (male, DSC), respectively (Fig. 7I, J) and all three of these groups showed significant increases in inner diameter at mean pressure (Fig. 7A, B). The large increase in inner diameter at mean pressure (~2.2x) in male ASC compared to female ASC (~1.8x) and male DSC (~1.2x) suggests that the aorta becomes susceptible to dilation only when *E*_*inc*_ exceeds a certain threshold value [23]. Our previous work in MU mice throughout aneurysm formation, progression, and failure showed that an initial increase in circumferential material stiffness may be an early indicator of aortic dilation and may predict disease outcomes such as lifespan [8].

Aortic dissection and/or rupture are mechanical failures of the aortic wall, which can be closely linked to disruptions in microstructural remodeling and compromised wall mechanics [8–10]. It is unclear whether microstructural remodeling and wall mechanics are independently associated with aortic dilation and/or failure or if they can affect it in a combined fashion. Our individual correlation analyses indicated that microstructural remodeling and wall mechanical metrics individually correlated with aortic dilation (Figs. 10 and 11). However, these individual results did not clarify the actual contribution of each metric and/or their interdependence. To address this, we used mixed regression models to evaluate multivariable contributions and rejected models with high multicollinearity. The mixed model analyses indicated that aortic dilation could be effectively predicted by combining three metrics from three different types of measurements: elastic fiber porosity from microstructural imaging, high modulus from passive mechanical tests, and unloaded wall thickness from unloaded dimension measurements (Fig. 12). Among these metrics, high modulus played the most significant role, contributing more prominently to the diameter prediction than the other two metrics.

The statistical analyses indicates that the three metrics in Model 3, elastic fiber porosity, high modulus, and unloaded wall thickness, are not highly colinear, but they are all related to aneurysm progression in MU mice and likely influence each other. Pores in the elastic fibers must be filled with something, likely additional collagen and deposition of proteoglycans [59,60], that will affect the high modulus and alter the unloaded thickness. We speculate that an increase in high modulus restricts aortic wall deformation, leading to localized stress on microstructural components. This triggers further microstructural remodeling [76], which subsequently alters wall mechanics and unloaded thickness. Increased wall thickness occurs through addition of mass that is mostly ECM. Because functional elastic fibers are typically not synthesized in adulthood [2], the deposited ECM is mostly collagen, with some proteoglycans that will further increase the high modulus and may exacerbate elastic fiber porosity under mechanical loading, resulting in aortic dilation. Single cell RNA-Seq analyses identified a unique population of modified SMCs present in dilated ascending aorta or aortic root from MU mice [70], a milder MFS mouse model (*Fbn1*^*C1041G/*+^), and humans with MFS [69]. The top 20 Reactome pathways enriched in the modified SMCs in all three types of aortic tissue included ECM organization and degradation, proteoglycans, and collagen formation, assembly, biosynthesis, degradation, and chain trimerization. The enriched pathways support the involvement of ECM remodeling that alters high modulus and wall thickness as a key factor in TAA associated with MFS. Overall, the multivariable analysis indicates that three of our metrics (elastic fiber porosity, high modulus, and unloaded wall thickness) can predict aortic dilation in MU mice and may be useful as physical biomarkers for aneurysm monitoring, prevention, and treatment.

### 4.5. Clinical implications

For patients with MFS, surgical intervention to replace the aortic root and ascending aorta is typically recommended when the aortic diameter reaches 4.5–5.0 cm, depending on age, rate of growth, family history, extent of the aorta included in the dilation, and the presence or absence of marked vertebral artery tortuosity. There are no specific recommendations regarding sex. Despite regular monitoring and these detailed recommendations, aortic root dilation and type A dissection are the primary cause of morbidity and mortality in MFS [12]. Aortic dissection and/or rupture occurs from biomechanical failure of the wall, hence monitoring biomechanical metrics and/or measures of ECM wall remodeling related to wall stiffness and strength along the aortic length, in addition to monitoring aortic diameter, and considering sex-specific differences, may improve clinical management of TAA in MFS. Our results suggest that elastic fiber porosity, unloaded wall thickness, and high modulus are predictive of aortic diameter, which is the primary clinical metric currently used for surgical decision making.

There are limited studies investigating elastic fiber porosity and unloaded thickness measurements in humans with MFS. High-frequency ultrasonic tissue anisotropy that correlates with microstructural disorganization showed much higher anisotropy in dilated MFS aorta compared to control [77] demonstrating clinical measures of microstructural remodeling (i.e. elastic fiber porosity) that may be useful for predicting aneurysmal dilation. A review of 15 studies reporting wall thickness and ascending aortic aneurysm diameter found no association between wall thickness and aortic dilation, but noted a need for consistent and clinically applicable methods to quantify wall thickness in ascending aortic aneurysm research [78]. Neither study separated their results based on sex. These examples demonstrate that imaging methods already used for aneurysm management in MFS may be able to provide additional information for clinical management. However, additional studies are needed to examine the role of elastic fiber porosity and unloaded wall thickness in aneurysmal dilation and outcomes in males and females with MFS.

While high modulus as defined in our study can only be measured in vitro, it is related to circumferential material stiffness of the wall at high blood pressure. High blood pressure could be induced during measurements of material stiffness using drugs or physical activity [75]. Circumferential incremental material stiffness, *E*_*inc*_, can be measured from dynamic imaging (such as ultrasound or MRI) that includes systolic and diastolic diameters and wall thickness, as well as pulse pressure (Eqn. 1b) [45]. PWV is a common clinical measure of circumferential structural stiffness that depends on the incremental material stiffness, diameter, and thickness of the wall, as well as the blood density [45]. Kröner et al. [16] measured PWV for five different aortic regions (ASC, aortic arch, DSC, SAB, and IAB) and observed increased PWV in MFS individuals compared to healthy controls for most regions. They also reported small, but significant aortic dilation for most aortic regions in MFS individuals. Guala et al. [31] observed increased PWV that correlated with dilation amounts in the ASC and DSC of individuals with MFS. Neither study separated their data based on sex. These clinical studies support our findings in the mouse and highlight the potential of circumferential aortic stiffness measures to predict aneurysm development and progression in MFS. Additional studies are needed to investigate the role of material versus structural stiffness and sex-specific effects in human aorta. Overall, our results suggest that multivariable measurements of different aneurysm properties may provide information about aortic dilation and disease progression.

### 4.6. Limitations and future work

Our data were collected from a mouse model of MFS and care must be taken in extrapolating results to human disease. Our results can suggest future studies in humans, such as evaluating sex- and region-specific outcomes and therapeutic treatments in MFS; however, further human studies are required to evaluate the usefulness of our suggested biomarkers in disease diagnosis, monitoring, and treatment. Due to the limited availability of human aortic samples, conducting extensive experiments is challenging. However, computational approaches can help overcome this limitation by utilizing patient-specific hemodynamic data, medical images and region-specific mechanical properties [79]. The number of samples/group in our study was limited by the expected lifespan of the MU mice, especially the males [8,80]. The relatively small sample size can affect robustness of the conclusions through reduced power of the statistical analyses. The microstructural remodeling results were determined from extensive quantification of imaging results. Future research should include biochemical, genetic, and proteomic confirmation of our observations. The passive mechanical testing results included a single inflation cycle at the physiologic axial stretch ratio. Additional research should include active mechanical testing and biaxial characterization with additional test protocols. We showed sex- and region-specific differences in microstructural remodeling, mechanical metrics, and unloaded dimensions, but we did not investigate mechanisms for the differences.

## 5. Conclusions

We investigated sex- and region-specific differences in microstructural remodeling and wall mechanical metrics and their relationship with aneurysmal dilation in a mouse model of severe MFS. The observed sex- and region-specific differences highlight the intricate process of wall remodeling that includes ECM and cellular degradation or deposition, wall stiffening, and wall thickening that drive differential susceptibility to aortic aneurysm formation in MFS. Our findings indicate that among the included metrics, a combination of microstructural (elastic fiber porosity), mechanical (high modulus), and unloaded dimension (thickness) metrics provides the highest predictive model for aneurysmal dilation, as quantified by the aortic inner diameter at mean pressure. The high modulus, a measure of material stiffness under high pressure, was the predominant contributor to the predictive model and demonstrates potential as a physical biomarker for the detection and management of aortic aneurysms associated with MFS.

## Supplementary Material

Supplementary data

Supplementary material associated with this article can be found, in the online version, at doi:10.1016/j.actbio.2025.05.056.

## Figures and Tables

**Table 1:** Average blood pressures of WT and MU, male and female mice. *N* = 5 – 10/group. Mean ± SD [8].

	Pressure (mmHg)	Systolic	Diastolic	Mean	Pulse
**Sex**	**Genotype**				
Male	WT	127 ± 6	96 ± 6	106 ± 6	31 ± 4
	MU	127 ± 4	94 ± 3	105 ± 3	32 ± 4
Female	WT	126 ± 7	93 ± 10	104 ± 9	33 ± 7
	MU	124 ± 12	96 ± 5	105 ± 7	28 ± 9

**Table 2:** Coefficients for each variable, *p*-value, and variance inflation factor (VIF) corresponding to the best (highest R^2^) model for each case for the multivariable regression.

	Coefficients for each variable						
	Elastic fiber porosity (ELP)	Nuclei count (NC)	Medial collagen content (CC_M)	Axial stress (AS)	High modulus (HM)	Unloaded thickness (UT)	Intercept
**Model 1**	10,800	−298	613	–	–	–	1440
**Model 2**	–	–	–	1.74	0.549	–	1440
**Model 3**	5020	–	–	–	0.360	11.1	1440
	p-value (VIF)						
	Elastic fiber porosity	Nuclei count	Medial collagen content	Axial stress	High modulus	Unloaded thickness	
**Model 1**	0.0001 (2.02)	<0.001 (1.11)	<0.001 (2.05)	–	–	–	
**Model 2**	–	–	–	0.0059 (1.18)	<0.001 (1.18)	–	
**Model 3**	0.0066 (2.17)	–	–	–	<0.001 (2.85)	<0.001 (1.61)	
