# Supplementary material for: Sex- and region-specific differences in microstructural remodeling and passive biomechanics of the aorta correlate with aneurysm propensity in a mouse model of severe Marfan syndrome
Source: Acta Biomater. Author manuscript; Available in PMC 2025 Sep 8. (PMC12416491; doi:10.1016/j.actbio.2025.05.056)
Supplement: Supplementary data [file NIHMS2105955-supplement-Supplementary_data.pdf]

Supplementary Figures and Tables

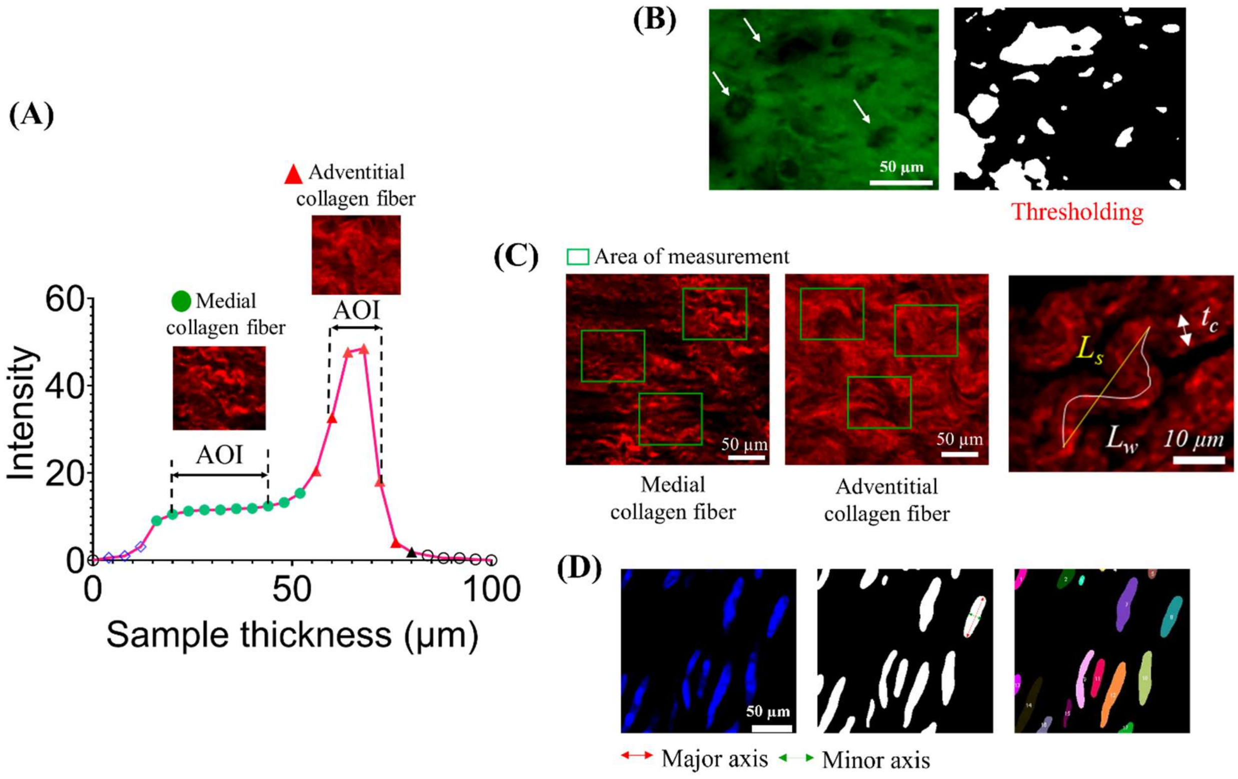

Supplemental Figure S1. The variation in collagen fiber intensity over the aortic wall thickness was used to determine the images covering the medial and adventitial layers of the aortic wall (A). Elastic fiber porosity (indicated by white arrows) was determined from the elastic fiber images in the medial region as the ratio of no signal (white) to total area of the images (B). Collagen fiber amount (normalized intensity of SHG signal), collagen fiber tortuosity (L_w/L_s) and thickness (t_c) (C) were determined from the collagen fiber images for the medial and adventitial layers. Cell nuclei number and aspect ratio (major/minor axis lengths) (D) were determined from the DAPI channel for the medial layer.

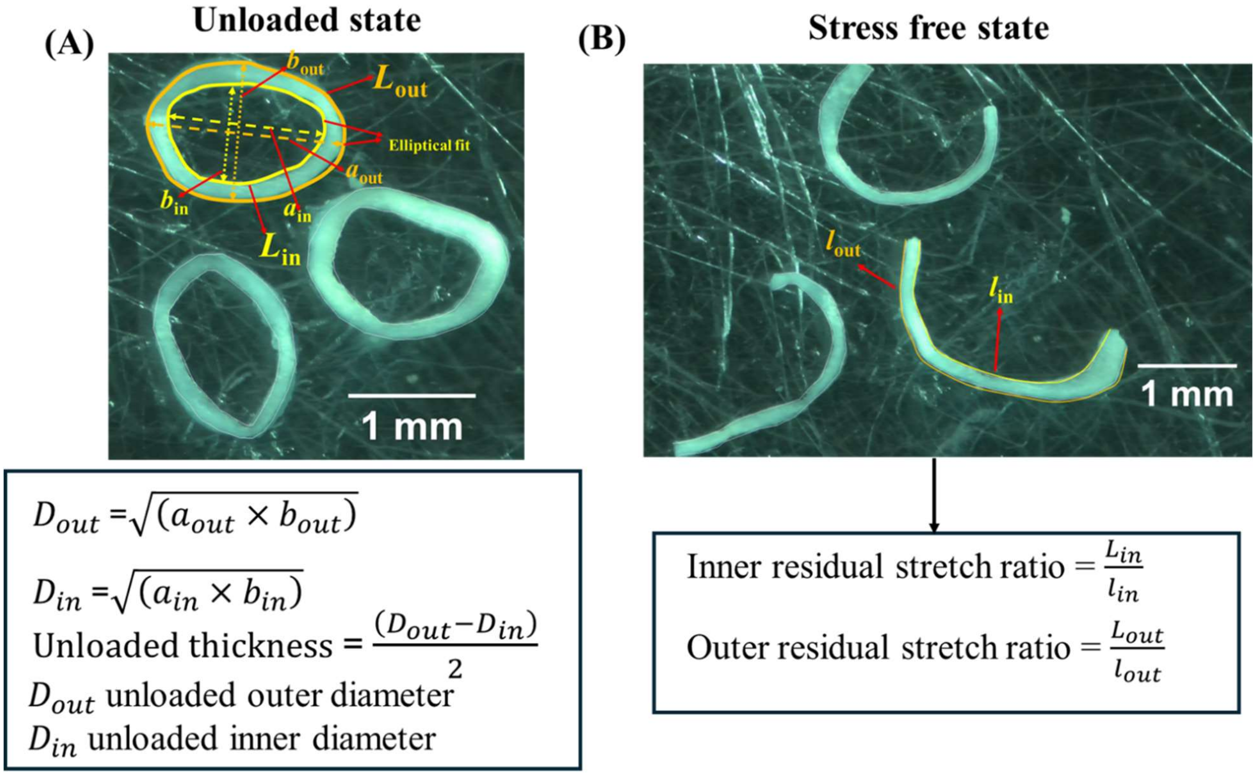

Supplemental Figure S2. Ellipses were fitted to the outer and inner perimeters of each unloaded aortic ring to determine the value of major (a_{out}; outer, a_{in}; inner) and minor (b_{out}; outer, b_{in}; inner) axes. These values of major and minor axis of ellipses were used to calculate the outer and inner unloaded diameters and wall thickness of each aortic ring (A). The dimensions from three aortic rings were averaged and used for subsequent measurements. The inner and outer perimeters (L_{in}, L_{out}) of the unloaded rings were also measured for residual stretch calculations. Inner and outer perimeters (l_{in}, l_{out}) of stress-free, open-cut rings were measured to assess residual stretches (B). ImageJ software (NIH) was used for all measurements.

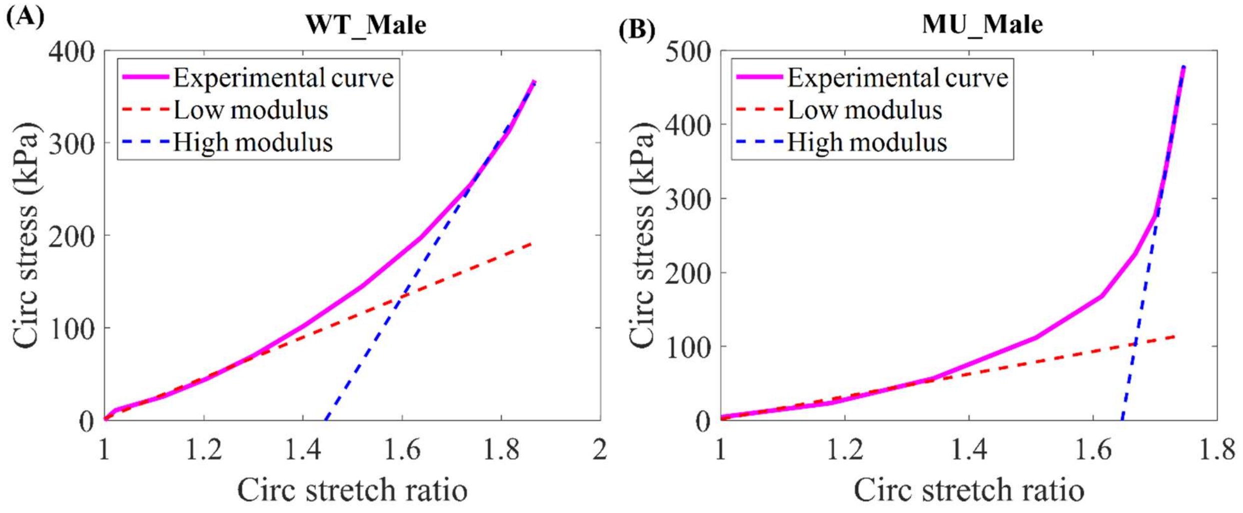

Supplemental Figure S3. Examples of low (lower stretch region) and high (higher stretch region) modulus fits to the circumferential stretch-stress curves of (A) WT and (B) MU male ASC.

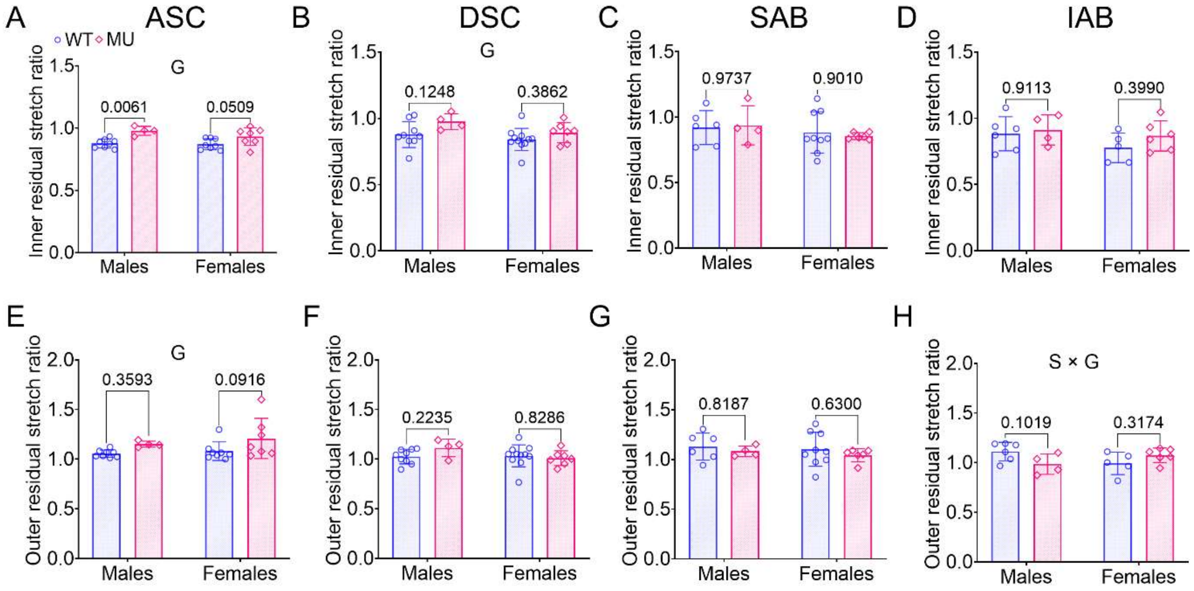

Supplemental Figure S4. Inner (A-D) and outer (E-H) residual stretch ratios of ASC, DSC, SAB, and IAB for each group. Residual stretch was measured from the ratio of inner/outer perimeters in the unloaded and stress-free configurations (Supp. Fig S2). Letters indicate significant effects by two-way ANOVA for independent variables (sex (S) and genotype (G)). P values indicates significant difference between genotype for each sex by Tukey's post hoc test. Individual data points and means ± SD are shown. N = 4 – 11/group. WT = blue circles, MU = pink diamonds.

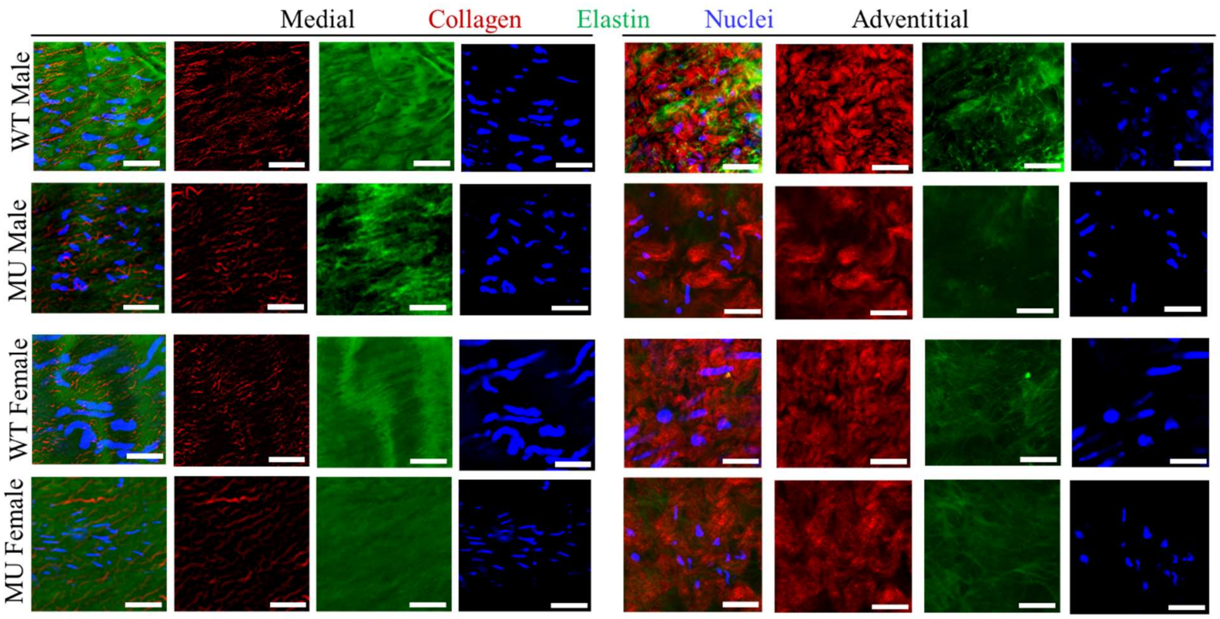

Supplemental Figure S5. Representative en face multiphoton images of DSC for WT and MU, male and female mice. These images illustrate the qualitative changes in collagen fiber (red), elastic fiber (green), and cell nuclei (blue) organization in the medial and adventitial layers for each group. Scale bars = 50 µm.

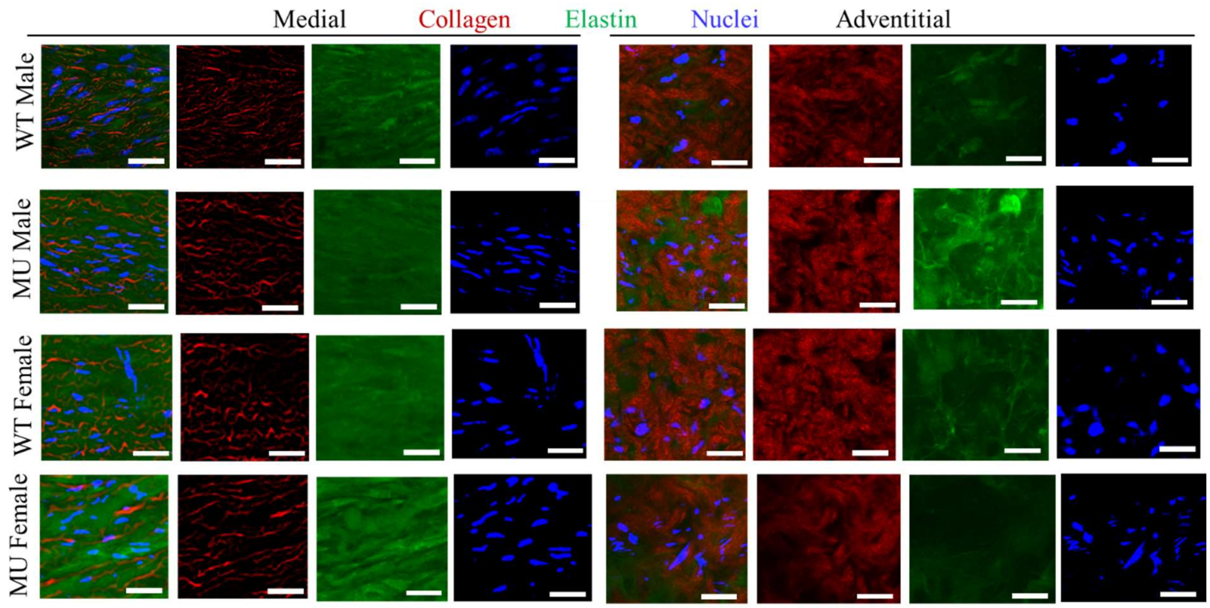

Supplemental Figure S6. Representative en face multiphoton images of SAB for WT and MU, male and female mice. These images illustrate the qualitative changes in collagen fiber (red), elastic fiber (green), and cell nuclei (blue) organization in the medial and adventitial layers for each group. Scale bars = 50 µm.

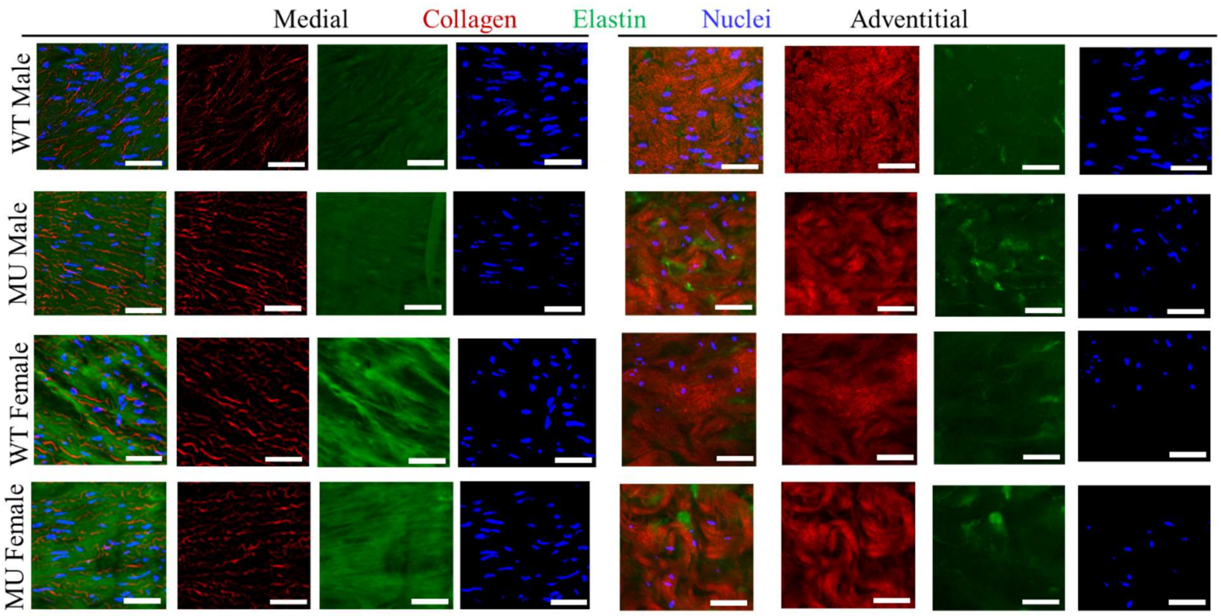

Supplemental Figure S7. Representative en face multiphoton images of IAB for WT and MU, male and female mice. These images illustrate the qualitative changes in collagen fiber (red), elastic fiber (green), and cell nuclei (blue) organization in the medial and adventitial layers for each group. Scale bars = 50 μm.

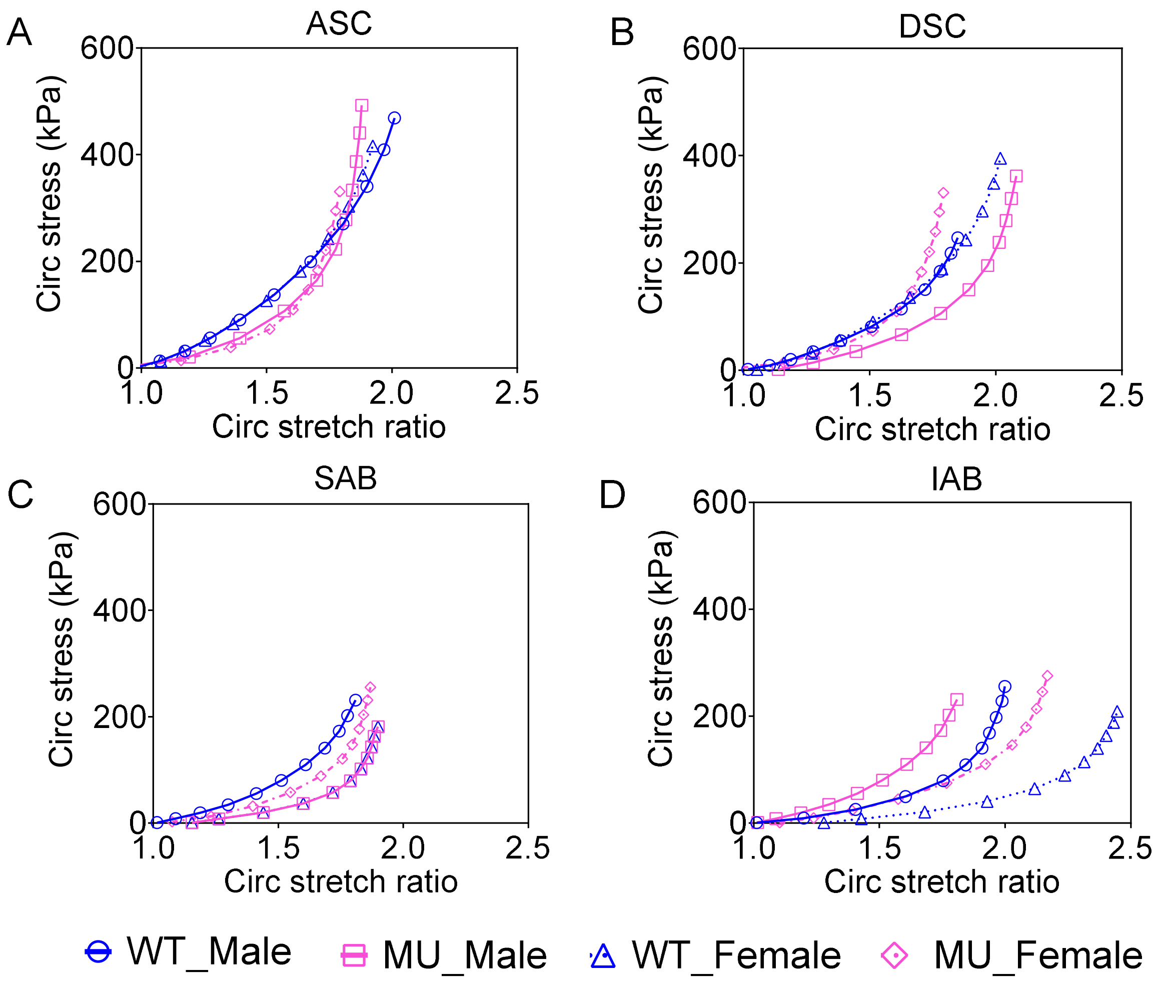

Supplemental Figure S8. Average circumferential stretch versus stress curves for ASC (A), DSC (B), SAB (C) and I AB (D). Circumferential stretch and stress were calculated using Eqns. 2a and 2b. Error bars are not shown for clarity. N = 5 – 12/group. WT Male = blue circles, MU male = pink squares. WT Female = blue triangles, MU Female = pink diamonds. Groupwise numbers of mice are presented in supplementary Table-1.

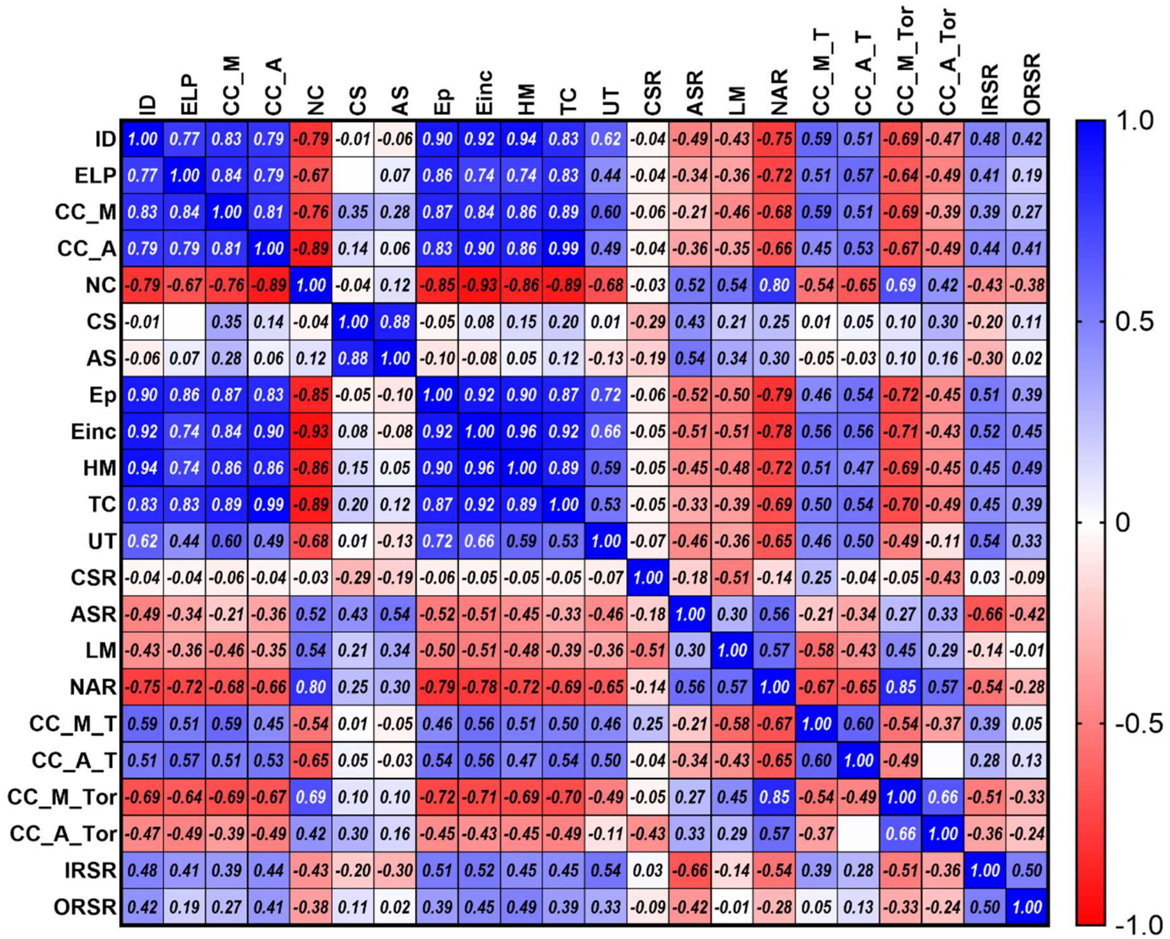

Supplemental Figure S9. Pearson correlation matrix for ASC. Numbers indicate the Pearson correlation coefficient, r. The corresponding p-value is given in Supp. Table S2. ID = inner diameter at mean pressure; ELP = elastic fiber porosity; CC_M = medial collagen content; CC_A = adventitial collagen content; NC = nuclei count; CS = circumferential stress; AS = axial stress; Ep = physiologic structural stiffness; Einc = physiologic material stiffness; HM = high modulus; TC = total collagen content; UT = unloaded thickness; CSR = circumferential stretch ratio; ASR = axial stretch ratio; LM = low modulus; NAR = nuclei aspect ratio; CC_M_T = medial collagen fiber thickness; CC_A_T = adventitial collagen fiber thickness; CC_M_Tor = medial collagen fiber tortuosity; CC_A_Tor = adventitial collagen fiber tortuosity; IRSR = inner residual stretch raio; ORSR = outer residual stretch ratio.

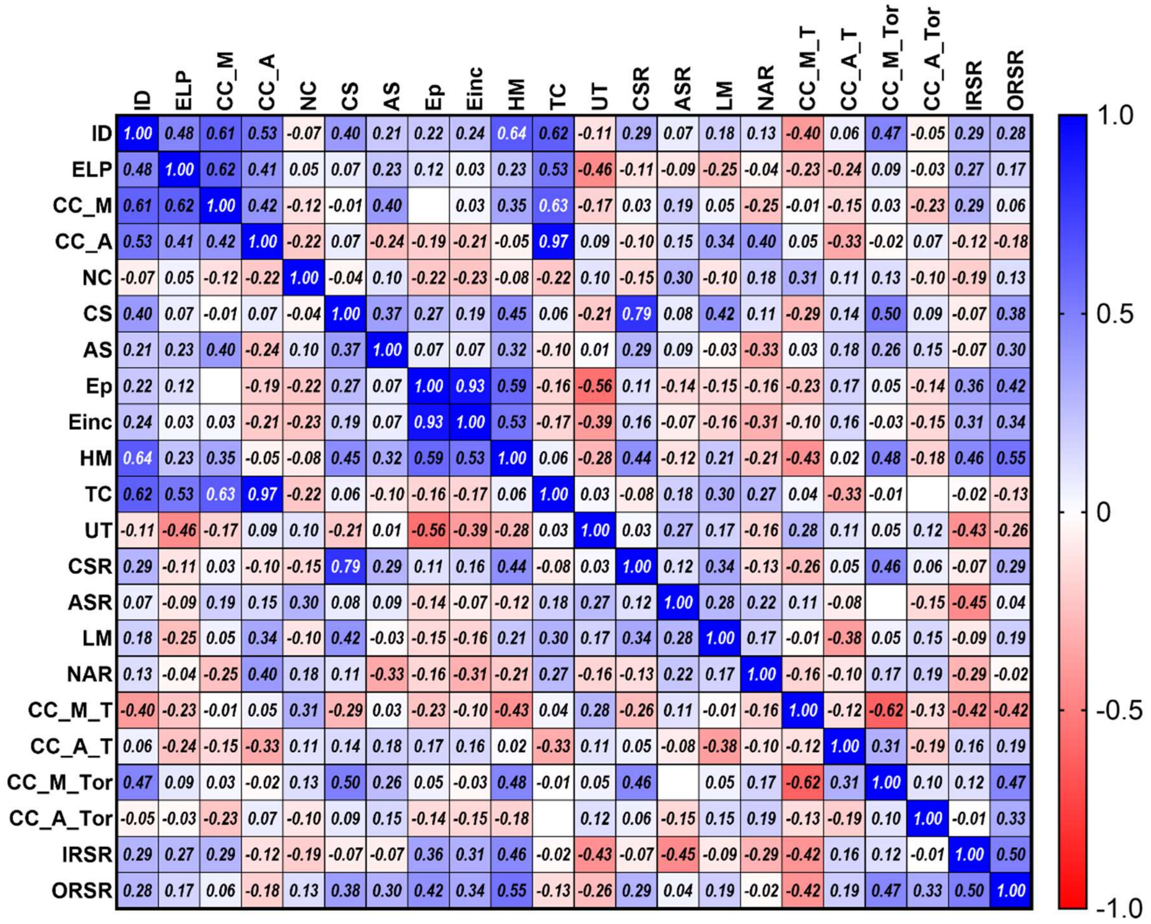

Supplemental Figure S10. Pearson correlation matrix for DSC. Numbers indicate the Pearson correlation coefficient, r. The corresponding p-value is given in Supp. Table S3. ID = inner diameter at mean pressure; ELP = elastic fiber porosity; CC_M = medial collagen content; CC_A = adventitial collagen content; NC = nuclei count; CS = circumferential stress; AS = axial stress; Ep = physiologic structural stiffness; Einc = physiologic material stiffness; HM = high modulus; TC = total collagen content; UT = unloaded thickness; CSR = circumferential stretch ratio; ASR = axial stretch ratio; LM = low modulus; NAR = nuclei aspect ratio; CC_M_T = medial collagen fiber thickness; CC_A_T = adventitial collagen fiber thickness; CC_M_Tor = medial collagen fiber tortuosity; CC_A_Tor = adventitial collagen fiber tortuosity; IRSR = inner residual stretch raio; ORSR = outer residual stretch ratio.

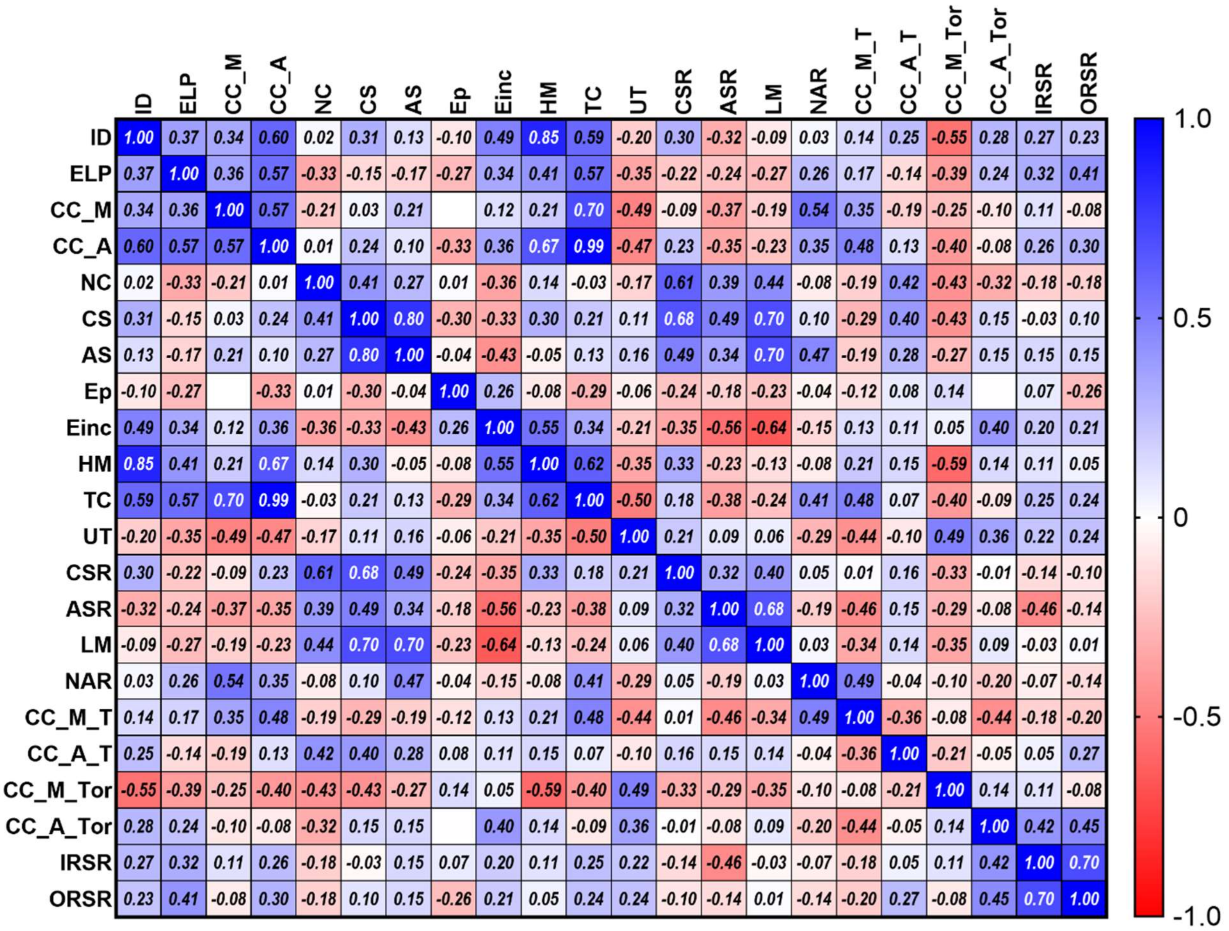

Supplemental Figure S11. Pearson correlation matrix for SAB. Numbers indicate the Pearson correlation coefficient, r. The corresponding p-value is given in Supp. Table S4. ID = inner diameter at mean pressure; ELP = elastic fiber porosity; CC_M = medial collagen content; CC_A = adventitial collagen content; NC = nuclei count; CS = circumferential stress; AS = axial stress; Ep = physiologic structural stiffness; Einc = physiologic material stiffness; HM = high modulus; TC = total collagen content; UT = unloaded thickness; CSR = circumferential stretch ratio; ASR = axial stretch ratio; LM = low modulus; NAR = nuclei aspect ratio; CC_M_T = medial collagen fiber thickness; CC_A_T = adventitial collagen fiber thickness; CC_M_Tor = medial collagen fiber tortuosity; CC_A_Tor = adventitial collagen fiber tortuosity; IRSR = inner residual stretch raio; ORSR = outer residual stretch ratio.

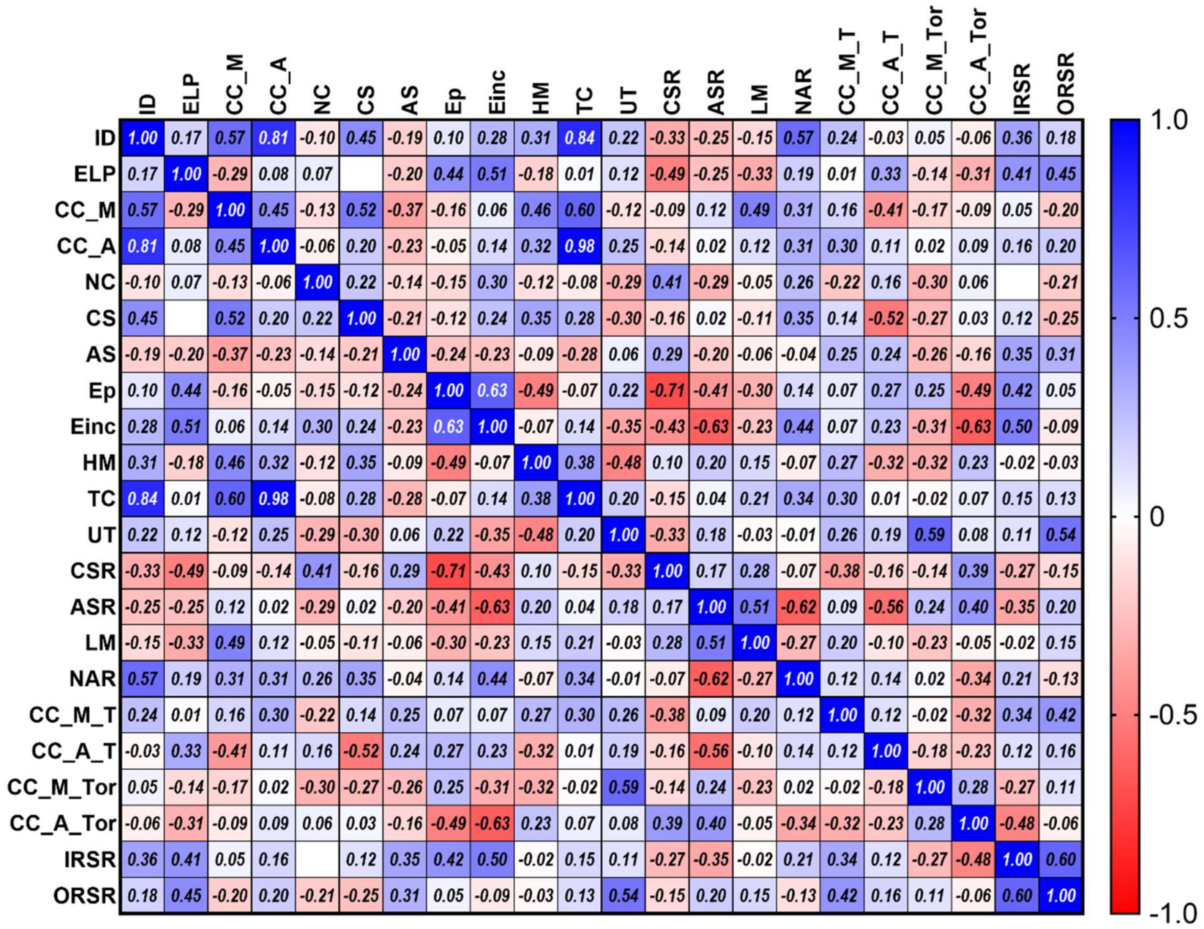

Supplemental Figure S12. Pearson correlation matrix for IAB. Numbers indicate the Pearson correlation coefficient, r. The corresponding p-value is given in Supp. Table S5. ID = inner diameter at mean pressure; ELP = elastic fiber porosity; CC_M = medial collagen content; CC_A = adventitial collagen content; NC = nuclei count; CS = circumferential stress; AS = axial stress; Ep = physiologic structural stiffness; Einc = physiologic material stiffness; HM = high modulus; TC = total collagen content; UT = unloaded thickness; CSR = circumferential stretch ratio; ASR = axial stretch ratio; LM = low modulus; NAR = nuclei aspect ratio; CC_M_T = medial collagen fiber thickness; CC_A_T = adventitial collagen fiber thickness; CC_M_Tor = medial collagen fiber tortuosity; CC_A_Tor = adventitial collagen fiber tortuosity; IRSR = inner residual stretch raio; ORSR = outer residual stretch ratio.

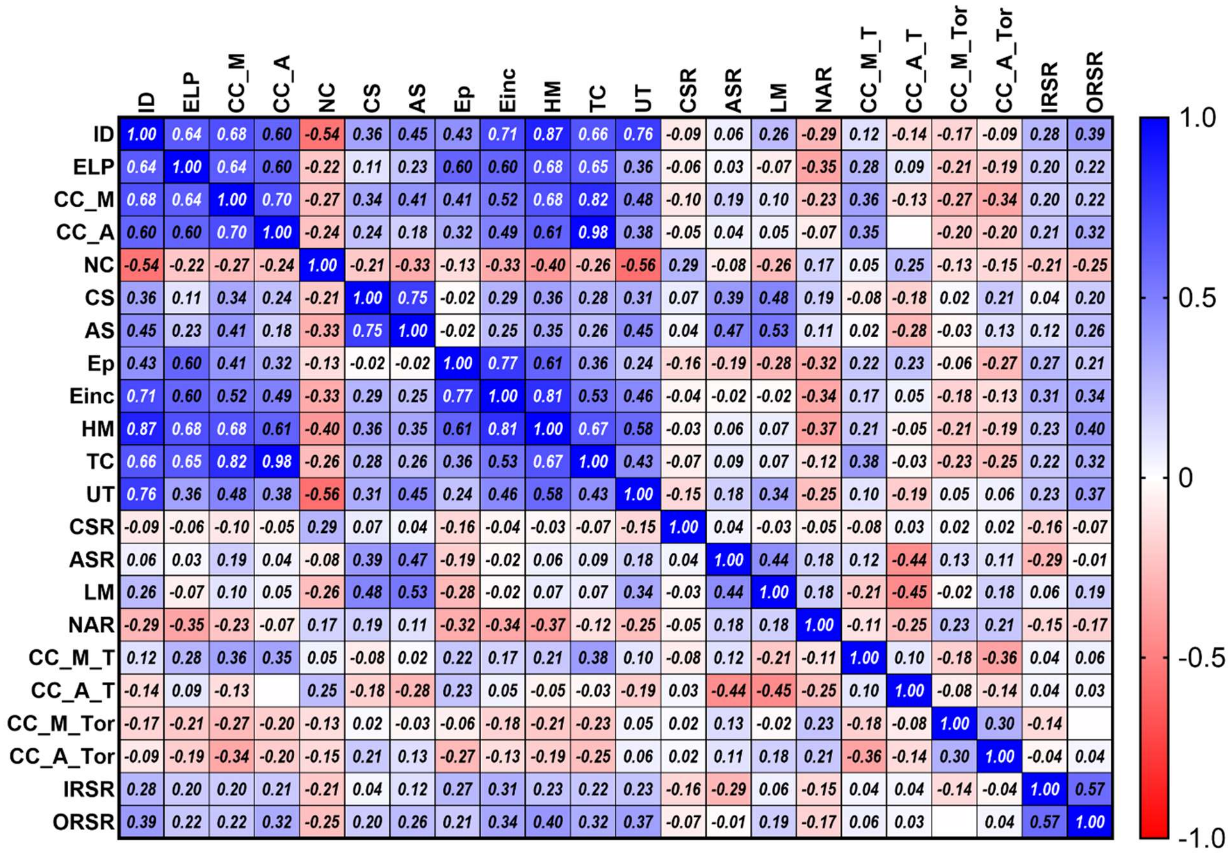

Supplemental Figure S13. Pearson correlation matrix for pooled data from all aortic segments. Numbers indicate the Pearson correlation coefficient, r. The corresponding p-value is given in Supp. Table S6. ID = inner diameter at mean pressure; ELP = elastic fiber porosity; CC_M = medial collagen content; CC_A = adventitial collagen content; NC = nuclei count; CS = circumferential stress; AS = axial stress; Ep = physiologic structural stiffness; Einc = physiologic material stiffness; HM = high modulus; TC = total collagen content; UT = unloaded thickness; CSR = circumferential stretch ratio; ASR = axial stretch ratio; LM = low modulus; NAR = nuclei aspect ratio; CC_M_T = medial collagen fiber thickness; CC_A_T = adventitial collagen fiber thickness; CC_M_Tor = medial collagen fiber tortuosity; CC_A_Tor = adventitial collagen fiber tortuosity; IRSR = inner residual stretch raio; ORSR = outer residual stretch ratio.

Supplemental Table S1. Number of samples for each measurement. ID = Inner diameter.

Sex	Male								Female							
**Genotype**	WT				MU				WT				MU			
**Aortic segment**	ASC	DSC	SAB	IAB	ASC	DSC	SAB	IAB	ASC	DSC	SAB	IAB	ASC	DSC	SAB	IAB
**Microstructural remodeling metrics**																
Elastic fiber porosity	6	6	6	6	5	5	5	5	6	6	6	6	6	6	6	6
Collagen measurements	6	6	6	6	5	5	5	5	6	6	6	6	6	6	6	6
Cell nuclei measurements	6	6	6	6	5	5	5	5	6	6	6	6	6	6	6	6
**Unloaded geometry and mechanical metrics**																
Unloaded ID	10	10	6	7	5	5	5	5	12	12	9	8	10	10	8	9
Unloaded thickness	10	10	6	7	5	5	5	5	12	12	9	8	10	10	8	9
ID at mean pressure	10	10	6	7	5	5	5	5	11	12	9	8	10	10	8	8
E_p	10	9	6	7	5	5	5	5	11	12	9	8	10	10	8	8
E_{inc}	10	9	6	7	5	5	5	5	11	12	9	8	10	10	8	7
Circ stretch	10	10	6	7	5	5	5	5	11	12	9	8	10	10	7	7
Axial stretch	10	10	6	7	5	5	5	5	11	12	9	8	10	10	7	7
Circ stress	10	10	6	7	5	5	5	5	11	12	9	8	10	10	7	7
Axial stress	10	10	6	7	5	5	5	5	11	12	9	8	10	10	7	7
Low modulus	10	10	6	7	5	5	5	5	9	11	9	8	9	10	7	6
High modulus	9	10	6	7	5	5	5	5	11	11	9	8	9	9	6	6

Supplemental Table S2. P values from the Pearson correlation analysis indicate significant correlation between variables for ASC. ID = inner diameter at mean pressure; ELP = elastic fiber porosity; CC_M = medial collagen content; CC_A = adventitial collagen content; NC = nuclei count; CS = circumferential stress; AS = axial stress; Ep = physiologic structural stiffness; Einc = physiologic material stiffness; HM = high modulus; TC = total collagen content; UT = unloaded thickness; CSR = circumferential stretch ratio; ASR = axial stretch ratio; LM = low modulus; NAR = nuclei aspect ratio; CC_M_T = medial collagen fiber thickness; CC_A_T = adventitial collagen fiber thickness; CC_M_Tor = medial collagen fiber tortuosity; CC_A_Tor = adventitial collagen fiber tortuosity; IRSR = inner residual stretch ratio; ORSR = outer residual stretch ratio.

ASC																						
	ID	ELP	CC_M	CC_A	NC	CS	AS	Ep	Einc	HM	TC	UT	CSR	ASR	LM	NAR	CC_M_T	CC_A_T	CC_M_Tor	CC_A_Tor	IRSR	ORSR
ID		0.00	0.00	0.00	0.00	0.98	0.79	0.00	0.00	0.00	0.00	0.00	0.87	0.03	0.06	0.00	0.01	0.02	0.00	0.04	0.03	0.06
ELP	0.00		0.00	0.00	0.00	0.99	0.77	0.00	0.00	0.00	0.00	0.05	0.86	0.14	0.12	0.00	0.02	0.01	0.00	0.03	0.08	0.41
CC_M	0.00	0.00		0.00	0.00	0.13	0.23	0.00	0.00	0.00	0.00	0.00	0.79	0.37	0.04	0.00	0.01	0.02	0.00	0.09	0.09	0.26
CC_A	0.00	0.00	0.00		0.00	0.56	0.80	0.00	0.00	0.00	0.00	0.03	0.87	0.12	0.13	0.00	0.05	0.02	0.00	0.03	0.05	0.07
NC	0.00	0.00	0.00	0.00		0.86	0.62	0.00	0.00	0.00	0.00	0.00	0.89	0.02	0.01	0.00	0.01	0.00	0.00	0.06	0.06	0.10
CS	0.98	0.99	0.13	0.56	0.86		0.00	0.85	0.73	0.53	0.41	0.96	0.22	0.06	0.37	0.28	0.98	0.85	0.66	0.20	0.39	0.64
AS	0.79	0.77	0.23	0.80	0.62	0.00		0.67	0.74	0.83	0.62	0.60	0.43	0.01	0.14	0.20	0.83	0.90	0.68	0.50	0.20	0.92
Ep	0.00	0.00	0.00	0.00	0.00	0.85	0.67		0.00	0.00	0.00	0.00	0.80	0.02	0.03	0.00	0.04	0.01	0.00	0.05	0.02	0.09
Einc	0.00	0.00	0.00	0.00	0.00	0.73	0.74	0.00		0.00	0.00	0.00	0.85	0.02	0.02	0.00	0.01	0.01	0.00	0.06	0.02	0.05
HM	0.00	0.00	0.00	0.00	0.00	0.53	0.83	0.00	0.00		0.00	0.01	0.83	0.05	0.03	0.00	0.02	0.03	0.00	0.04	0.05	0.03
TC	0.00	0.00	0.00	0.00	0.00	0.41	0.62	0.00	0.00	0.00		0.02	0.85	0.15	0.09	0.00	0.03	0.01	0.00	0.03	0.05	0.09
UT	0.00	0.05	0.00	0.03	0.00	0.96	0.60	0.00	0.00	0.01	0.02		0.78	0.04	0.12	0.00	0.04	0.03	0.03	0.64	0.01	0.15
CSR	0.87	0.86	0.79	0.87	0.89	0.22	0.43	0.80	0.85	0.83	0.85	0.78		0.44	0.02	0.55	0.29	0.86	0.84	0.06	0.91	0.71
ASR	0.03	0.14	0.37	0.12	0.02	0.06	0.01	0.02	0.02	0.05	0.15	0.04	0.44		0.21	0.01	0.38	0.14	0.26	0.15	0.00	0.07
LM	0.06	0.12	0.04	0.13	0.01	0.37	0.14	0.03	0.02	0.03	0.09	0.12	0.02	0.21		0.01	0.01	0.06	0.05	0.21	0.55	0.97
NAR	0.00	0.00	0.00	0.00	0.00	0.28	0.20	0.00	0.00	0.00	0.00	0.00	0.55	0.01	0.01		0.00	0.00	0.00	0.01	0.01	0.22
CC_M_T	0.01	0.02	0.01	0.05	0.01	0.98	0.83	0.04	0.01	0.02	0.03	0.04	0.29	0.38	0.01	0.00		0.01	0.01	0.11	0.09	0.84
CC_A_T	0.02	0.01	0.02	0.02	0.00	0.85	0.90	0.01	0.01	0.03	0.01	0.03	0.86	0.14	0.06	0.00	0.01		0.03	0.98	0.22	0.59
CC_M_Tor	0.00	0.00	0.00	0.00	0.00	0.66	0.68	0.00	0.00	0.00	0.00	0.03	0.84	0.26	0.05	0.00	0.01	0.03		0.00	0.02	0.15
CC_A_Tor	0.04	0.03	0.09	0.03	0.06	0.20	0.50	0.05	0.06	0.04	0.03	0.64	0.06	0.15	0.21	0.01	0.11	0.98	0.00		0.12	0.30
IRSR	0.03	0.08	0.09	0.05	0.06	0.39	0.20	0.02	0.02	0.05	0.05	0.01	0.91	0.00	0.55	0.01	0.09	0.22	0.02	0.12		0.02
ORSR	0.06	0.41	0.26	0.07	0.10	0.64	0.92	0.09	0.05	0.03	0.09	0.15	0.71	0.07	0.97	0.22	0.84	0.59	0.15	0.30	0.02	

Supplemental Table S3. P values from the Pearson correlation analysis indicate significant correlation between variables for DSC. ID = inner diameter at mean pressure; ELP = elastic fiber porosity; CC_M = medial collagen content; CC_A = adventitial collagen content; NC = nuclei count; CS = circumferential stress; AS = axial stress; Ep = physiologic structural stiffness; Einc = physiologic material stiffness; HM = high modulus; TC = total collagen content; UT = unloaded thickness; CSR = circumferential stretch ratio; ASR = axial stretch ratio; LM = low modulus; NAR = nuclei aspect ratio; CC_M_T = medial collagen fiber thickness; CC_A_T = adventitial collagen fiber thickness; CC_M_Tor = medial collagen fiber tortuosity; CC_A_Tor = adventitial collagen fiber tortuosity; IRSR = inner residual stretch ratio; ORSR = outer residual stretch ratio.

DSC																						
	ID	ELP	CC_M	CC_A	NC	CS	AS	Ep	Einc	HM	TC	UT	CSR	ASR	LM	NAR	CC_M_T	CC_A_T	CC_M_Tor	CC_A_Tor	IRSR	ORSR
ID		0.03	0.00	0.02	0.78	0.08	0.37	0.36	0.32	0.00	0.00	0.63	0.21	0.76	0.44	0.58	0.08	0.80	0.03	0.84	0.22	0.23
ELP	0.03		0.00	0.07	0.82	0.77	0.33	0.61	0.89	0.32	0.02	0.04	0.65	0.69	0.28	0.88	0.33	0.31	0.70	0.91	0.25	0.48
CC_M	0.00	0.00		0.07	0.60	0.98	0.08	0.99	0.89	0.13	0.00	0.46	0.91	0.42	0.84	0.29	0.97	0.53	0.89	0.34	0.22	0.80
CC_A	0.02	0.07	0.07		0.36	0.76	0.30	0.42	0.39	0.84	0.00	0.71	0.68	0.52	0.15	0.08	0.85	0.15	0.93	0.77	0.61	0.45
NC	0.78	0.82	0.60	0.36		0.86	0.67	0.35	0.33	0.74	0.36	0.66	0.53	0.20	0.67	0.45	0.19	0.65	0.59	0.69	0.42	0.58
CS	0.08	0.77	0.98	0.76	0.86		0.11	0.26	0.42	0.05	0.80	0.37	0.00	0.74	0.07	0.64	0.22	0.55	0.03	0.71	0.76	0.10
AS	0.37	0.33	0.08	0.30	0.67	0.11		0.77	0.78	0.17	0.69	0.97	0.21	0.72	0.91	0.15	0.91	0.45	0.26	0.53	0.78	0.19
Ep	0.36	0.61	0.99	0.42	0.35	0.26	0.77		0.00	0.01	0.50	0.01	0.63	0.55	0.54	0.51	0.33	0.48	0.84	0.56	0.12	0.06
Einc	0.32	0.89	0.89	0.39	0.33	0.42	0.78	0.00		0.02	0.49	0.09	0.49	0.77	0.49	0.19	0.67	0.51	0.90	0.52	0.19	0.14
HM	0.00	0.32	0.13	0.84	0.74	0.05	0.17	0.01	0.02		0.81	0.23	0.05	0.63	0.37	0.37	0.06	0.94	0.03	0.46	0.04	0.01
TC	0.00	0.02	0.00	0.00	0.36	0.80	0.69	0.50	0.49	0.81		0.91	0.75	0.44	0.20	0.25	0.88	0.16	0.97	0.99	0.92	0.57
UT	0.63	0.04	0.46	0.71	0.66	0.37	0.97	0.01	0.09	0.23	0.91		0.90	0.26	0.46	0.49	0.23	0.65	0.84	0.61	0.06	0.27
CSR	0.21	0.65	0.91	0.68	0.53	0.00	0.21	0.63	0.49	0.05	0.75	0.90		0.62	0.14	0.58	0.27	0.83	0.04	0.80	0.76	0.21
ASR	0.76	0.69	0.42	0.52	0.20	0.74	0.72	0.55	0.77	0.63	0.44	0.26	0.62		0.24	0.36	0.64	0.75	1.00	0.52	0.04	0.85
LM	0.44	0.28	0.84	0.15	0.67	0.07	0.91	0.54	0.49	0.37	0.20	0.46	0.14	0.24		0.47	0.96	0.10	0.84	0.54	0.72	0.43
NAR	0.58	0.88	0.29	0.08	0.45	0.64	0.15	0.51	0.19	0.37	0.25	0.49	0.58	0.36	0.47		0.50	0.68	0.48	0.43	0.21	0.92
CC_M_T	0.08	0.33	0.97	0.85	0.19	0.22	0.91	0.33	0.67	0.06	0.88	0.23	0.27	0.64	0.96	0.50		0.62	0.00	0.58	0.07	0.06
CC_A_T	0.80	0.31	0.53	0.15	0.65	0.55	0.45	0.48	0.51	0.94	0.16	0.65	0.83	0.75	0.10	0.68	0.62		0.19	0.41	0.49	0.41
CC_M_Tor	0.03	0.70	0.89	0.93	0.59	0.03	0.26	0.84	0.90	0.03	0.97	0.84	0.04	1.00	0.84	0.48	0.00	0.19		0.67	0.61	0.04
CC_A_Tor	0.84	0.91	0.34	0.77	0.69	0.71	0.53	0.56	0.52	0.46	0.99	0.61	0.80	0.52	0.54	0.43	0.58	0.41	0.67		0.96	0.16
IRSR	0.22	0.25	0.22	0.61	0.42	0.76	0.78	0.12	0.19	0.04	0.92	0.06	0.76	0.04	0.72	0.21	0.07	0.49	0.61	0.96		0.03
ORSR	0.23	0.48	0.80	0.45	0.58	0.10	0.19	0.06	0.14	0.01	0.57	0.27	0.21	0.85	0.43	0.92	0.06	0.41	0.04	0.16	0.03	

Supplemental Table S4. P values from the Pearson correlation analysis indicate significant correlation between variables for SAB. ID = inner diameter at mean pressure; ELP = elastic fiber porosity; CC_M = medial collagen content; CC_A = adventitial collagen content; NC = nuclei count; CS = circumferential stress; AS = axial stress; Ep = physiologic structural stiffness; Einc = physiologic material stiffness; HM = high modulus; TC = total collagen content; UT = unloaded thickness; CSR = circumferential stretch ratio; ASR = axial stretch ratio; LM = low modulus; NAR = nuclei aspect ratio; CC_M_T = medial collagen fiber thickness; CC_A_T = adventitial collagen fiber thickness; CC_M_Tor = medial collagen fiber tortuosity; CC_A_Tor = adventitial collagen fiber tortuosity; IRSR = inner residual stretch ratio; ORSR = outer residual stretch ratio.

SAB																						
	ID	ELP	CC_M	CC_A	NC	CS	AS	Ep	Einc	HM	TC	UT	CSR	ASR	LM	NAR	CC_M_T	CC_A_T	CC_M_Tor	CC_A_Tor	IRSR	ORSR
ID		0.37	0.34	0.60	0.02	0.31	0.13	-0.10	0.49	0.85	0.59	-0.20	0.30	-0.32	-0.09	0.03	0.14	0.25	-0.55	0.28	0.27	0.23
ELP	0.37		0.36	0.57	-0.33	-0.15	-0.17	-0.27	0.34	0.41	0.57	-0.35	-0.22	-0.24	-0.27	0.26	0.17	-0.14	-0.39	0.24	0.32	0.41
CC_M	0.34	0.36		0.57	-0.21	0.03	0.21	0.00	0.12	0.21	0.70	-0.49	-0.09	-0.37	-0.19	0.54	0.35	-0.19	-0.25	-0.10	0.11	-0.08
CC_A	0.60	0.57	0.57		0.01	0.24	0.10	-0.33	0.36	0.67	0.99	-0.47	0.23	-0.35	-0.23	0.35	0.48	0.13	-0.40	-0.08	0.26	0.30
NC	0.02	-0.33	-0.21	0.01		0.41	0.27	0.01	-0.36	0.14	-0.03	-0.17	0.61	0.39	0.44	-0.08	-0.19	0.42	-0.43	-0.32	-0.18	-0.18
CS	0.31	-0.15	0.03	0.24	0.41		0.80	-0.30	-0.33	0.30	0.21	0.11	0.68	0.49	0.70	0.10	-0.29	0.40	-0.43	0.15	-0.03	0.10
AS	0.13	-0.17	0.21	0.10	0.27	0.80		-0.04	-0.43	-0.05	0.13	0.16	0.49	0.34	0.70	0.47	-0.19	0.28	-0.27	0.15	0.15	0.15
Ep	-0.10	-0.27	0.00	-0.33	0.01	-0.30	-0.04		0.26	-0.08	-0.29	-0.06	-0.24	-0.18	-0.23	-0.04	-0.12	0.08	0.14	0.00	0.07	-0.26
Einc	0.49	0.34	0.12	0.36	-0.36	-0.33	-0.43	0.26		0.55	0.34	-0.21	-0.35	-0.56	-0.64	-0.15	0.13	0.11	0.05	0.40	0.20	0.21
HM	0.85	0.41	0.21	0.67	0.14	0.30	-0.05	-0.08	0.55		0.62	-0.35	0.33	-0.23	-0.13	-0.08	0.21	0.15	-0.59	0.14	0.11	0.05
TC	0.59	0.57	0.70	0.99	-0.03	0.21	0.13	-0.29	0.34	0.62		-0.50	0.18	-0.38	-0.24	0.41	0.48	0.07	-0.40	-0.09	0.25	0.24
UT	-0.20	-0.35	-0.49	-0.47	-0.17	0.11	0.16	-0.06	-0.21	-0.35	-0.50		0.21	0.09	0.06	-0.29	-0.44	-0.10	0.49	0.36	0.22	0.24
CSR	0.30	-0.22	-0.09	0.23	0.61	0.68	0.49	-0.24	-0.35	0.33	0.18	0.21		0.32	0.40	0.05	0.01	0.16	-0.33	-0.01	-0.14	-0.10
ASR	-0.32	-0.24	-0.37	-0.35	0.39	0.49	0.34	-0.18	-0.56	-0.23	-0.38	0.09	0.32		0.68	-0.19	-0.46	0.15	-0.29	-0.08	-0.46	-0.14
LM	-0.09	-0.27	-0.19	-0.23	0.44	0.70	0.70	-0.23	-0.64	-0.13	-0.24	0.06	0.40	0.68		0.03	-0.34	0.14	-0.35	0.09	-0.03	0.01
NAR	0.03	0.26	0.54	0.35	-0.08	0.10	0.47	-0.04	-0.15	-0.08	0.41	-0.29	0.05	-0.19	0.03		0.49	-0.04	-0.10	-0.20	-0.07	-0.14
CC_M_T	0.14	0.17	0.35	0.48	-0.19	-0.29	-0.19	-0.12	0.13	0.21	0.48	-0.44	0.01	-0.46	-0.34	0.49		-0.36	-0.08	-0.44	-0.18	-0.20
CC_A_T	0.25	-0.14	-0.19	0.13	0.42	0.40	0.28	0.08	0.11	0.15	0.07	-0.10	0.16	0.15	0.14	-0.04	-0.36		-0.21	-0.05	0.05	0.27
CC_M_Tor	-0.55	-0.39	-0.25	-0.40	-0.43	-0.43	-0.27	0.14	0.05	-0.59	-0.40	0.49	-0.33	-0.29	-0.35	-0.10	-0.08	-0.21		0.14	0.11	-0.08
CC_A_Tor	0.28	0.24	-0.10	-0.08	-0.32	0.15	0.15	0.00	0.40	0.14	-0.09	0.36	-0.01	-0.08	0.09	-0.20	-0.44	-0.05	0.14		0.42	0.45
IRSR	0.27	0.32	0.11	0.26	-0.18	-0.03	0.15	0.07	0.20	0.11	0.25	0.22	-0.14	-0.46	-0.03	-0.07	-0.18	0.05	0.11	0.42		0.70
ORSR	0.23	0.41	-0.08	0.30	-0.18	0.10	0.15	-0.26	0.21	0.05	0.24	0.24	-0.10	-0.14	0.01	-0.14	-0.20	0.27	-0.08	0.45	0.70	

Supplemental Table S5. P values from the Pearson correlation analysis indicate significant correlation between variables for IAB. ID = inner diameter at mean pressure; ELP = elastic fiber porosity; CC_M = medial collagen content; CC_A = adventitial collagen content; NC = nuclei count; CS = circumferential stress; AS = axial stress; Ep = physiologic structural stiffness; Einc = physiologic material stiffness; HM = high modulus; TC = total collagen content; UT = unloaded thickness; CSR = circumferential stretch ratio; ASR = axial stretch ratio; LM = low modulus; NAR = nuclei aspect ratio; CC_M_T = medial collagen fiber thickness; CC_A_T = adventitial collagen fiber thickness; CC_M_Tor = medial collagen fiber tortuosity; CC_A_Tor = adventitial collagen fiber tortuosity; IRSR = inner residual stretch ratio; ORSR = outer residual stretch ratio.

IAB																						
	ID	ELP	CC_M	CC_A	NC	CS	AS	Ep	Einc	HM	TC	UT	CSR	ASR	LM	NAR	CC_M_T	CC_A_T	CC_M_Tor	CC_A_Tor	IRSR	ORSR
ID		0.17	0.57	0.81	-0.10	0.45	-0.19	0.10	0.28	0.31	0.84	0.22	-0.33	-0.25	-0.15	0.57	0.24	-0.03	0.05	-0.06	0.36	0.18
ELP	0.17		-0.29	0.08	0.07	0.00	-0.20	0.44	0.51	-0.18	0.01	0.12	-0.49	-0.25	-0.33	0.19	0.01	0.33	-0.14	-0.31	0.41	0.45
CC_M	0.57	-0.29		0.45	-0.13	0.52	-0.37	-0.16	0.06	0.46	0.60	-0.12	-0.09	0.12	0.49	0.31	0.16	-0.41	-0.17	-0.09	0.05	-0.20
CC_A	0.81	0.08	0.45		-0.06	0.20	-0.23	-0.05	0.14	0.32	0.98	0.25	-0.14	0.02	0.12	0.31	0.30	0.11	0.02	0.09	0.16	0.20
NC	-0.10	0.07	-0.13	-0.06		0.22	-0.14	-0.15	0.30	-0.12	-0.08	-0.29	0.41	-0.29	-0.05	0.26	-0.22	0.16	-0.30	0.06	0.00	-0.21
CS	0.45	0.00	0.52	0.20	0.22		-0.21	-0.12	0.24	0.35	0.28	-0.30	-0.16	0.02	-0.11	0.35	0.14	-0.52	-0.27	0.03	0.12	-0.25
AS	-0.19	-0.20	-0.37	-0.23	-0.14	-0.21		-0.24	-0.23	-0.09	-0.28	0.06	0.29	-0.20	-0.06	-0.04	0.25	0.24	-0.26	-0.16	0.35	0.31
Ep	0.10	0.44	-0.16	-0.05	-0.15	-0.12	-0.24		0.63	-0.49	-0.07	0.22	-0.71	-0.41	-0.30	0.14	0.07	0.27	0.25	-0.49	0.42	0.05
Einc	0.28	0.51	0.06	0.14	0.30	0.24	-0.23	0.63		-0.07	0.14	-0.35	-0.43	-0.63	-0.23	0.44	0.07	0.23	-0.31	-0.63	0.50	-0.09
HM	0.31	-0.18	0.46	0.32	-0.12	0.35	-0.09	-0.49	-0.07		0.38	-0.48	0.10	0.20	0.15	-0.07	0.27	-0.32	-0.32	0.23	-0.02	-0.03
TC	0.84	0.01	0.60	0.98	-0.08	0.28	-0.28	-0.07	0.14	0.38		0.20	-0.15	0.04	0.21	0.34	0.30	0.01	-0.02	0.07	0.15	0.13
UT	0.22	0.12	-0.12	0.25	-0.29	-0.30	0.06	0.22	-0.35	-0.48	0.20		-0.33	0.18	-0.03	-0.01	0.26	0.19	0.59	0.08	0.11	0.54
CSR	-0.33	-0.49	-0.09	-0.14	0.41	-0.16	0.29	-0.71	-0.43	0.10	-0.15	-0.33		0.17	0.28	-0.07	-0.38	-0.16	-0.14	0.39	-0.27	-0.15
ASR	-0.25	-0.25	0.12	0.02	-0.29	0.02	-0.20	-0.41	-0.63	0.20	0.04	0.18	0.17		0.51	-0.62	0.09	-0.56	0.24	0.40	-0.35	0.20
LM	-0.15	-0.33	0.49	0.12	-0.05	-0.11	-0.06	-0.30	-0.23	0.15	0.21	-0.03	0.28	0.51		-0.27	0.20	-0.10	-0.23	-0.05	-0.02	0.15
NAR	0.57	0.19	0.31	0.31	0.26	0.35	-0.04	0.14	0.44	-0.07	0.34	-0.01	-0.07	-0.62	-0.27		0.12	0.14	0.02	-0.34	0.21	-0.13
CC_M_T	0.24	0.01	0.16	0.30	-0.22	0.14	0.25	0.07	0.07	0.27	0.30	0.26	-0.38	0.09	0.20	0.12		0.12	-0.02	-0.32	0.34	0.42
CC_A_T	-0.03	0.33	-0.41	0.11	0.16	-0.52	0.24	0.27	0.23	-0.32	0.01	0.19	-0.16	-0.56	-0.10	0.14	0.12		-0.18	-0.23	0.12	0.16
CC_M_Tor	0.05	-0.14	-0.17	0.02	-0.30	-0.27	-0.26	0.25	-0.31	-0.32	-0.02	0.59	-0.14	0.24	-0.23	0.02	-0.02	-0.18		0.28	-0.27	0.11
CC_A_Tor	-0.06	-0.31	-0.09	0.09	0.06	0.03	-0.16	-0.49	-0.63	0.23	0.07	0.08	0.39	0.40	-0.05	-0.34	-0.32	-0.23	0.28		-0.48	-0.06
IRSR	0.36	0.41	0.05	0.16	0.00	0.12	0.35	0.42	0.50	-0.02	0.15	0.11	-0.27	-0.35	-0.02	0.21	0.34	0.12	-0.27	-0.48		0.60
ORSR	0.18	0.45	-0.20	0.20	-0.21	-0.25	0.31	0.05	-0.09	-0.03	0.13	0.54	-0.15	0.20	0.15	-0.13	0.42	0.16	0.11	-0.06	0.60	

Supplemental Table S6. P values from the Pearson correlation analysis indicate significant correlation between variables for pooled data for all aortic segments. ID = inner diameter at mean pressure; ELP = elastic fiber porosity; CC_M = medial collagen content; CC_A = adventitial collagen content; NC = nuclei count; CS = circumferential stress; AS = axial stress; Ep = physiologic structural stiffness; Einc = physiologic material stiffness; HM = high modulus; TC = total collagen content; UT = unloaded thickness; CSR = circumferential stretch ratio; ASR = axial stretch ratio; LM = low modulus; NAR = nuclei aspect ratio; CC_M_T = medial collagen fiber thickness; CC_A_T = adventitial collagen fiber thickness; CC_M_Tor = medial collagen fiber tortuosity; CC_A_Tor = adventitial collagen fiber tortuosity; IRSR = inner residual stretch ratio; ORSR = outer residual stretch ratio. P-values < 0.05 are highlighted in orange.

	ID	ELP	CC_M	CC_A	NC	CS	AS	Ep	Einc	HM	TC	UT	CSR	ASR	LM	NAR	CC_M_T	CC_A_T	CC_M_Tor	CC_A_Tor	IRSR	ORSR
ID		0.00	0.00	0.00	0.00	0.00	0.00	0.00	0.00	0.00	0.00	0.00	0.45	0.61	0.02	0.01	0.31	0.22	0.16	0.46	0.01	0.00
ELP	0.00		0.00	0.00	0.05	0.37	0.05	0.00	0.00	0.00	0.00	0.00	0.63	0.82	0.57	0.00	0.01	0.43	0.07	0.10	0.08	0.06
CC_M	0.00	0.00		0.00	0.02	0.00	0.00	0.00	0.00	0.00	0.00	0.00	0.41	0.10	0.38	0.04	0.00	0.26	0.02	0.00	0.09	0.06
CC_A	0.00	0.00	0.00		0.04	0.04	0.12	0.01	0.00	0.00	0.00	0.00	0.67	0.71	0.65	0.56	0.00	0.98	0.09	0.09	0.07	0.00
NC	0.00	0.05	0.02	0.04		0.06	0.00	0.25	0.00	0.00	0.02	0.00	0.01	0.50	0.02	0.14	0.68	0.03	0.26	0.21	0.07	0.03
CS	0.00	0.37	0.00	0.04	0.06		0.00	0.90	0.01	0.00	0.01	0.01	0.54	0.00	0.00	0.11	0.49	0.13	0.86	0.07	0.74	0.09
AS	0.00	0.05	0.00	0.12	0.00	0.00		0.89	0.03	0.00	0.03	0.00	0.75	0.00	0.00	0.33	0.89	0.02	0.77	0.28	0.29	0.02
Ep	0.00	0.00	0.00	0.01	0.25	0.90	0.89		0.00	0.00	0.00	0.04	0.18	0.10	0.02	0.00	0.06	0.04	0.60	0.02	0.02	0.08
Einc	0.00	0.00	0.00	0.00	0.00	0.01	0.03	0.00		0.00	0.00	0.00	0.72	0.85	0.88	0.00	0.15	0.64	0.13	0.27	0.01	0.00
HM	0.00	0.00	0.00	0.00	0.00	0.00	0.00	0.00	0.00		0.00	0.00	0.80	0.60	0.54	0.00	0.06	0.66	0.07	0.10	0.04	0.00
TC	0.00	0.00	0.00	0.00	0.02	0.01	0.03	0.00	0.00	0.00		0.00	0.58	0.46	0.55	0.32	0.00	0.77	0.05	0.03	0.06	0.01
UT	0.00	0.00	0.00	0.00	0.00	0.01	0.00	0.04	0.00	0.00	0.00		0.20	0.12	0.00	0.03	0.38	0.10	0.70	0.64	0.05	0.00
CSR	0.45	0.63	0.41	0.67	0.01	0.54	0.75	0.18	0.72	0.80	0.58	0.20		0.73	0.80	0.65	0.51	0.77	0.88	0.86	0.18	0.56
ASR	0.61	0.82	0.10	0.71	0.50	0.00	0.00	0.10	0.85	0.60	0.46	0.12	0.73		0.00	0.13	0.30	0.00	0.25	0.36	0.01	0.91
LM	0.02	0.57	0.38	0.65	0.02	0.00	0.00	0.02	0.88	0.54	0.55	0.00	0.80	0.00		0.12	0.07	0.00	0.86	0.12	0.60	0.11
NAR	0.01	0.00	0.04	0.56	0.14	0.11	0.33	0.00	0.00	0.00	0.32	0.03	0.65	0.13	0.12		0.36	0.03	0.05	0.06	0.19	0.14
CC_M_T	0.31	0.01	0.00	0.00	0.68	0.49	0.89	0.06	0.15	0.06	0.00	0.38	0.51	0.30	0.07	0.36		0.41	0.11	0.00	0.75	0.60
CC_A_T	0.22	0.43	0.26	0.98	0.03	0.13	0.02	0.04	0.64	0.66	0.77	0.10	0.77	0.00	0.00	0.03	0.41		0.50	0.23	0.75	0.81
CC_M_Tor	0.16	0.07	0.02	0.09	0.26	0.86	0.77	0.60	0.13	0.07	0.05	0.70	0.88	0.25	0.86	0.05	0.11	0.50		0.01	0.22	0.98
CC_A_Tor	0.46	0.10	0.00	0.09	0.21	0.07	0.28	0.02	0.27	0.10	0.03	0.64	0.86	0.36	0.12	0.06	0.00	0.23	0.01		0.76	0.74
IRSR	0.01	0.08	0.09	0.07	0.07	0.74	0.29	0.02	0.01	0.04	0.06	0.05	0.18	0.01	0.60	0.19	0.75	0.75	0.22	0.76		0.00
ORSR	0.00	0.06	0.06	0.00	0.03	0.09	0.02	0.08	0.00	0.00	0.01	0.00	0.56	0.91	0.11	0.14	0.60	0.81	0.98	0.74	0.00	
